# Seasonality of antimicrobial resistance rates in respiratory bacteria: A systematic review and meta-analysis

**DOI:** 10.1371/journal.pone.0221133

**Published:** 2019-08-15

**Authors:** Evelyn Pamela Martinez, Magda Cepeda, Marija Jovanoska, Wichor M. Bramer, Josje Schoufour, Marija Glisic, Annelies Verbon, Oscar H. Franco

**Affiliations:** 1 Facultad de Medicina Veterinaria y Zootecnia, Universidad Central del Ecuador, Quito, Ecuador; 2 Department of Microbiology and Infectious Diseases, Erasmus MC, University Medical Centre, Rotterdam, the Netherlands; 3 Department of Epidemiology, Erasmus MC, University Medical Centre, Rotterdam, the Netherlands; 4 Medical Faculty, Saints Cyril and Methodius University of Skopje, Skopje, Macedonia; 5 Medical Library, Erasmus MC, University Medical Centre, Rotterdam, the Netherlands; 6 Leibniz Institute for Prevention Research and Epidemiology—BIPS, Bremen, Germany; 7 Institute of Social and Preventive Medicine, Faculty of Medicine, University of Bern, Bern, Switzerland; Rabin Medical Center, Beilinson Hospital, ISRAEL

## Abstract

**Background:**

Antimicrobial resistance (AMR) rates may display seasonal variation. However, it is not clear whether this seasonality is influenced by the seasonal variation of infectious diseases, geographical region or differences in antibiotic prescription patterns. Therefore, we assessed the seasonality of AMR rates in respiratory bacteria.

**Methods:**

Seven electronic databases (Embase.com, Medline Ovid, Cochrane CENTRAL, Web of Science, Core Collection, Biosis Ovid, and Google Scholar), were searched for relevant studies from inception to Jun 25^th^, 2019. Studies describing resistance rates of *Streptococcus pneumoniae* and *Haemophilus influenzae* were included in this review. By using random-effects meta-analysis, pooled odd ratios of seasonal AMR rates were calculated using winter as the reference group. Pooled odd ratios were obtained by antibiotic class and geographical region.

**Results:**

We included 13 studies, of which 7 were meta-analyzed. Few studies were done in *H*. *influenzae*, thus this was not quantitively analyzed. AMR rates of *S*. *pneumoniae* to penicillins were lower in other seasons than in winter with pooled OR = 0.71; 95% CI = 0.65–0.77; I^2^ = 0.0%, and to all antibiotics with pooled OR = 0.68; 95% CI = 0.60–0.76; I^2^ = 14.4%. Irrespective of geographical region, the seasonality of AMR rates in *S*. *pneumoniae* remained the same.

**Conclusion:**

The seasonality of AMR rates could result from the seasonality of infectious diseases and its accompanied antibiotic use.

## Introduction

Globally, bacterial respiratory infections are a leading cause of morbidity and mortality. *Streptococcus pneumoniae* and *Haemophilus influenzae* are a common cause of community-acquire pneumoniae and meningitis in children worldwide [1]. In 2015, pneumonia killed 920,136 children under 5 years, and accounted for 16% of all deaths in this age group [2]. In the last two decades, respiratory bacteria have increasingly becoming resistant to several antibiotics, and the prevalence of resistant strains is growing rapidly. Between the 20% to 30% of all pneumonias are caused by multidrug-resistant *S*. *pneumoniae* [1], and about the 30% to 40% are caused by penicillin-resistant *S*. *pneumoniae* [3–5]. Resistant infections lead to a longer stay in hospital, higher health-care costs, and increased mortality [6, 7]. In 2017, the World Health Organization (WHO) included *S*. *pneumoniae* and *H*. *influenza*e on the list of priority bacteria for which new antibiotics are needed [8].

Emerging evidence suggests that AMR rates in respiratory bacteria show seasonal variation as a results of a dynamic interaction between host and environment, and antibiotic selective pressure; however, results are highly variable among studies. For instance, prescription rates of penicillins, cephalosporins and macrolides increased by 75% and 100% in the winter compared to summer [9], which was associated with winter-peaks of resistance in *S*. *pneumoniae* to penicillins and cephalosporins [4, 10–12], whereas AMR rates to macrolides showed no seasonal variation [13]. A study in the United States showed that the rates of penicillin-resistant *S*. *pneumoniae* were higher in spring than in winter [5], while other studies in Spain showed higher resistance rates in both summer and winter [3, 13]. On the other hand, studies done in Israel and Lituania reported higher resistance rates of multidrug-resistant *S*. *pneumoniae* in winter than in summer [14, 15]. Furthermore, resistant rates of *H*. *influenzae* to penicillins and macrolides tended to be higher in winter than in summer in a study done in Japan [16], while another study from Italy did not find significant differences in resistance rates between autumn and spring [17].

Although, the variability in the seasonality of AMR rates has been linked to seasonal variation of antibiotic consumption [18–20], other factors such as different patterns of antibiotic use or geographical region may influence on the variation of AMR rates [9, 18]. In this era of increasing trends of AMR, it is pivotal to understand all phenomena contributing to the selection of AMR. Thus, we summarized relevant published studies to assess the seasonality of AMR rates in respiratory bacteria, and to identify factors underlying this pattern.

## Materials and methods

This systematic review and meta-analysis follows the guidelines in the Preferred Reporting Items for Systematic Reviews and Meta-Analysis (PRISMA) Statement (S1 Table) [21].

### Search strategy and selection criteria

The search strategy in this systemtaic review was originally established to find studies addressing the variation of AMR rates per month and/or per season in five bacteria included on the OMS priority bacteria list [8]: *Campylobacter spp*., *Salmonella spp*., *Escherichia coli*, *Streptococcus pneumoniae*, and *Haemophilus influenzae*. However, we decided to focus only on respiratoy bacteria, because studies in *E*. *coli*, *Salmonella spp*. *and Campylobacter spp*. where not eligible for further analysis due to either few number of available studies or high heterogeneity making it difficult to compare them.

Relevant studies were searched in seven electronic databases (Embase.com, Medline Ovid, Cochrane CENTRAL, Web of Science, Core Collection, Biosis Ovid, and Google Scholar) from inception until October 23^rd^, 2018, with an update until Jun 25^th^, 2019. The search terms list is shown in the supporting information (S1 Text). Studies reporting AMR rates in respiratory bacteria from at least two different seasons were eligible for consideration. We included studies that fulfilled the following inclusion criteria: 1) being cross-sectional, cohort, time-series, and longitudinal studies in design; 2) describing bacteria isolates from humans; 3) measuring antibiotic resistance as the study goal; 4) standard methods used for antibiotic susceptibility testing; 5) primary outcome reported per month or season.

### Study selection and quality assessment

Titles were retrieved from each electronic database and duplicates removed. The review of retrieved studies was done based on the method of Bramer *et al*. [22]. Briefly, independently, four authors (EPM, MG, MJ, JS) screened the title and abstracts of the retrieved references. Selected references were full-text retrieved and assessed for inclusion eligibility independently by two authors (EPM, MJ). A third author (MC) was available to discuss disagreements. For additional studies, the reference lists of included studies were hand-searched and corresponding authors of selected studies were contacted via e-mail.

As there is no single recommended tool for assessing the quality of cross-sectional studies, we developed a modified version of the Newcastle-Ottawa Scale (NOS) for cohort and case-control studies [23]. We used five quality criteria: 1) the representativeness of sample source; 2) the length of follow-up; 3) comparability of the population within the study period; 4) reliability measure of antibiotic sensitivity; and 5) a clear description of the outcome (S2 Text).

### Data extraction and analysis

Data was extracted from text, tables or graphs from each selected study; and was recorded in a customized form for this systematic review. We extracted data regarding authors, year of publication, study location, and duration, study design, sample size, characteristics of the study population and season definition. Also, we extracted the frequency or percentage of resistance to any antibiotic class (including multi-resistance profiles) per season (or monthly) and per bacterial species.

Studies were categorized according to the WHO regions definition as follows: Europe (Spain, Israel, Lithuania, England, Italy and Norway), America (The United States and Costa Rica), South East Asia (Thailand and India), and Western Pacific (Japan and Australia). Based on the mechanism of action, antibiotics were categorized into the following classes: penicillins, cephalosporins, macrolides, sulphamides and trimethoprim. Also, multidrug-resistance categorization was used to define isolates resistant to more than two antibiotic classes.

Meteorological seasons were defined as winter (December to February), summer (June to August), autumn (September to October), and spring (March to May) for studies in the northern hemisphere, whereas for studies in the southern hemisphere the definition of seasons was opposite. For studies that present results with other season definitions such as: wet (December to April) and dry (May to November), and as cold (October to March), and warm (April to September) were not considered for quantitative analyses.

For the quantitative analyses, we calculated for each study the seasonal resistance rates according to antibiotic class and geographical region. For this calculation, we used as the numerator the number of resistant isolates, and as the denominator, the total number of isolates tested per season. monthly resistance rates per year were provided, in this case resistance rates were pooled per month followed by season. In two studies [10, 11] the denominator was not available and this was imputed assuming the best-case scenario where the sampling was equally distributed across the length of the study. This assumption was based on the hypothesis that the proportion of resistance isolates would vary according to the season independently of the sample size. Thus, we calculated the denominator by dividing the overall number of isolates tested by the number of seasons included in the study (e.g. dividing by four if the study presented resistance rates in four seasons).

Secondly, we calculated separate seasonal resistance ratios according to antibiotic class using winter as the reference group. In an extra analyses, we compared spring and autumn in to include studies that only used these seasons, here spring was the reference group.

Finally, we carried out random-effects meta-analyses weighted by study size to calculate pooled odds ratios (OR) and 95% confidence intervals for each comparison stratified by antibiotic class and geographical region. Heterogeneity across studies was measured using the I^2^ test. We examined publication bias for each comparison by examining asymmetry funnel plots and using Egger's test. All tests were two-tailed and p-values < 0.05 were significant. Also, we performed sensitivity analyses by doing extra meta-analysis estimating pooled OR excluding studies carried on in countries located in different hemispheres, and those studies in which the denominator was imputed. All analyses were done using the statistical software STATA MP 14, and graphs were done in R 3.5.1 and R Studio 1.1.383.

## Results

### Description of included studies

**Fig 1:**
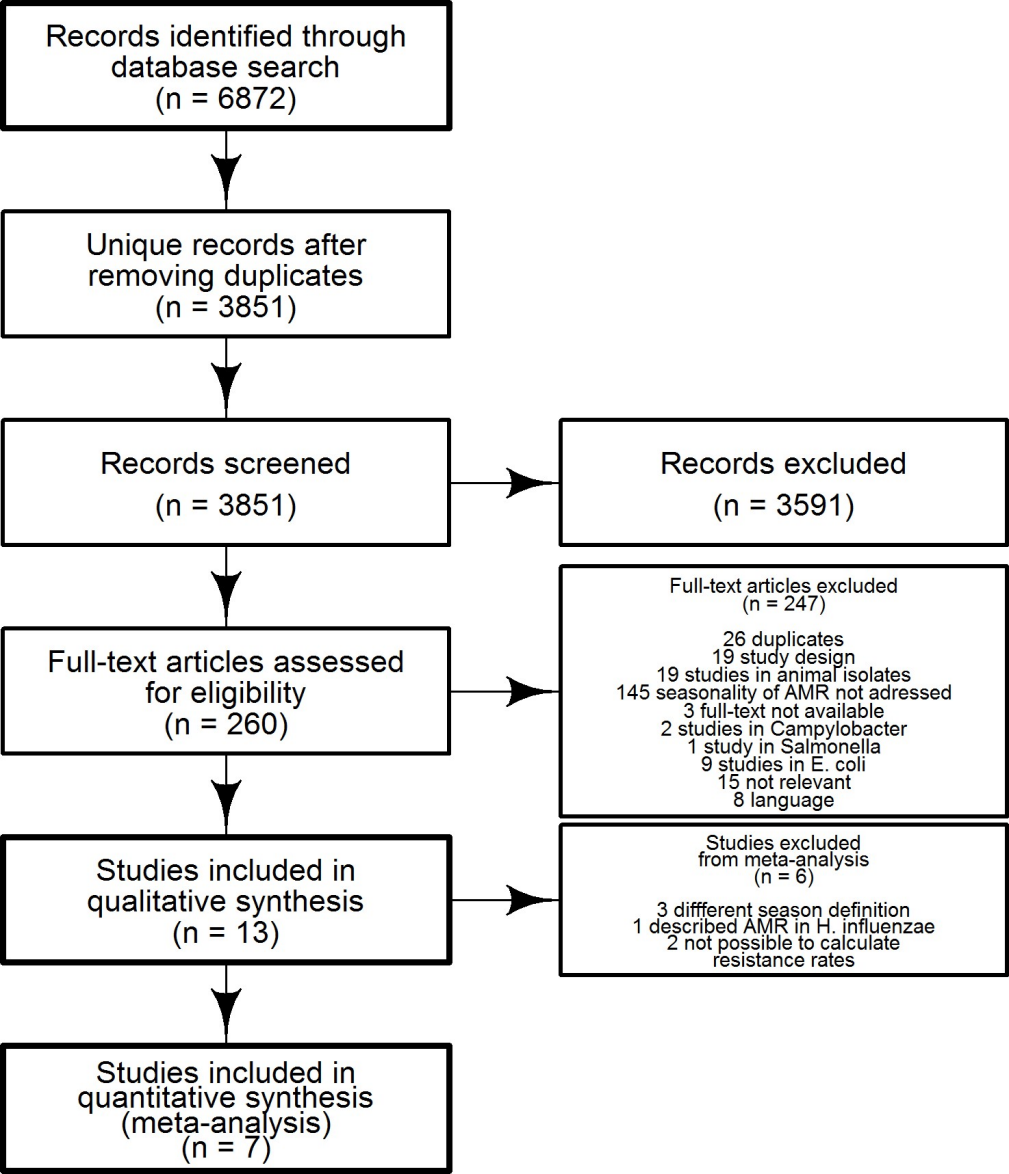
PRISMA flowchart summarizing the study selection process.

We retrieved 3.851 unique references, of which 258 studies were full-text assessed, and 13 studies were included for the qualitative synthesis, of which seven were meta-analyzed (Fig 1). No additional studies were identified by authors contact or hand-search nor after updating the literature search.

Most of the studies were cross-sectional (n = 7), performed in countries located in the northern hemisphere (n = 10), and in European countries (n = 6). The majority of studies were done for *S*. *pneumoniae* (n = 11), and on isolates from community-acquired infections in children with acute otitis media (n = 9). The most common resistance profile studied was against penicillins in *S*. *pneumoniae* isolates (n = 6) (Table 1).

**Table 1:** General descriptive information of included studies (n = 13).

Reference	Publication year	Study type	Country	Hemisphere	Study region	Study period	Sample source	AMR breakpoint	Season definition	AMR pattern
***Streptococcus pneumoniae***										
Albanese et al.[12]	2002	Cross-sectional	USA	N	AM	Jan 1995 to Dec 1997	Patients with respiratory infections	CSLI	2	PEN
Baquero et al. [13, 25]	1999	Prospective	Spain	N	EU	May 1996 to Apr 1997	Patients with respiratory infections	CSLI	1	PEN
Boken et al. [25]	1995	Cross-sectional	USA	N	AM	April to August 1994	Children aged 2 to 24 months with respiratory infections	CSLI	2	PEN
Dagan et al. [14]	2008	Prospective	Israel	N/E	EU	From 1998 to 2003	Children with acute otitis media	CSLI	3	PEN, CEP, MC, MDR
Guevara et al. [24]	2008	Cross-sectional	Costa Rica	N/W	AM	From 1994 to 2004	Children until 2 years with otitis media	CSLI	4	PEN, MC
Hoberman et al. [5]	2005	Cross-sectional	USA	N	AM	May 1991 to Apr 2003	Children 2 months to 7 years with respiratory infection	CSLI	1	PEN, MC, TM/SUL
Marco et al. [3]	2000	Cross-sectional	Spain	N	EU	May 1996 to Apr 1997	Patients with respiratory infections	CSLI	1	PEN, CEP, MC
Siripongpreeda et al. [4]	2010	Retrospective	Thailand	N/E	SEA	Jan 1997 to Dec 2007	Patients aged <18 with respiratory infection	CSLI	5	PEN
Stacevičiene et al. [15]	2016	Prospective	Lithuania	N	EU	Feb 2012 to Mar 2013	Children aged <6 years with respiratory infection	EUCAST	1	MDR
Tam et al. [10]	2015	Cross-sectional	USA	N	AM	From 2007 to 2012	Children aged < 5 years with respiratory infection	N/A	1	PEN
Vardhan & Allen [11]	2003	Prospective	England	N	EU	Jan 1987 to Dec 2000	Children with respiratory infection	CSLI	1	PEN
***Streptococcus pneumoniae and Haemophilus influenzae***										
Marchisio et al. [17]	2001	Longitudinal	Italy	N	EU	Oct–Nov in 1996, and Apr–May in 1997	Healthy children aged 1 to 7 years.	CSLI	2	PEN, MC
***Haemophilus influenzae***										
Hashida et al. [16]	2008	Cross-sectional	Japan	N	WP	Jul 2004 to Feb 2005	Healthy children aged 1 to 6 years	CSLI	5	PEN

N = Northern hemisphere, N/W = Northern/Western Hemisphere and N/E = Northern/Eastern hemisphere. EU = Europe, SEA = South-East Asia, WP = Western Pacific, and AM = Americas. 1 = Winter, Spring, Summer and Autumn, 2 = Spring vs Autumn, 3 = Cold vs Warm, 4 = Wet vs Dry, 5 = Winter vs Summer. CSLI = Clinical Laboratory Standards Institute, EUCAST = European Committee on Antibiotic Susceptibility Testing. PEN = penicillins, CEP = Cephalosporines, MC = Macrolides, SUL = Sulphamides, TM = Trimethoprim, and MDR = Multidrug-resistant. N/A = not available.

### Study quality

Quality assessment showed that most of included studies had fair quality (S2 Table). 11 (84.6%) studies were adjusted to control for confounders, and 12 (92.3%) studies used standard methods to measure the outcome of study. The common reason to score fair quality was the study time period, six studies (46.2%) did not allow us to categorize the four metereological seasons (S2 Table).

### Seasonality of antimicrobial resistance rates

Only two studies reported seasonality of AMR rates in *H*. *influenzae* [16, 17], thus they were not meta-analyzed. One study showed higher AMR rates to penicillins in spring than in autumn (10.1% vs 5.8%), whereas in the other study no significant differences in AMR rates to ampicillin between summer and winter were found (18.5% vs 16.8%; P>0.05).

Three studies reported AMR rates in *S*. *pneumoniae* with different season categorizations: wet vs dry [24], cold vs warm [14], and respiratory (i.e. winter and spring) vs non-respiratory seasons (i.e. summer and autumn) [12], and therefore were not meta-analyzed. These studies showed similar results as the meta-analyzed studies; higher AMR rates of penicillin-resistant isolates in the wet, respiratory season, and cold months (S3 Table).

**Fig 2:**
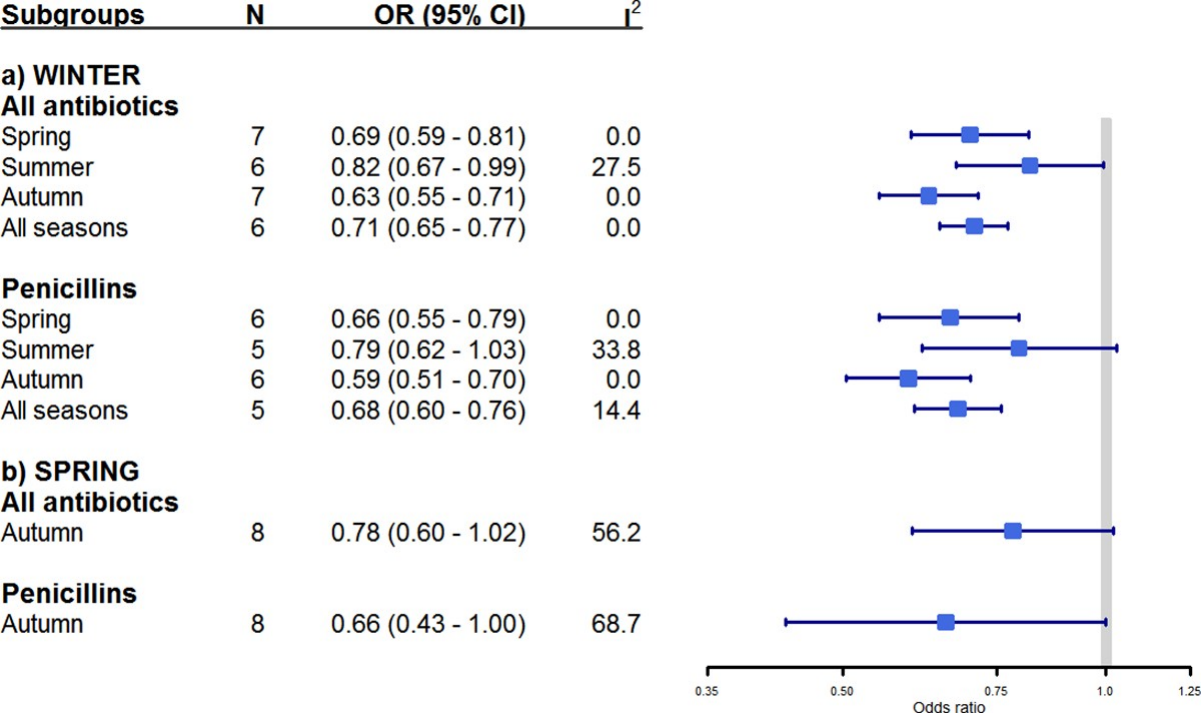
Forest plot of seasonality of antimicrobial resistance rates in *Streptococcus pneumoniae* isolates. Studies were stratified into two subgroups of antibiotics and estimates of effect are presented as pooled odds ratios (squares) with 95% confidence intervals (lateral lines of squares). For comparison, winter and spring were the reference groups, thus equal to one. Solid vertical line limits no difference between the two groups. I^2^ refers to percentage of heterogeneity among studies. The “All antibiotics” subgroup includes penicillins, cephalosporins, macrolides, trimethoprim/sulphamides and multi-drug resistance.

In the seven studies [3–5, 10, 11, 13, 15] used for the meta-analysis, AMR rates of *S*. *pneumoniae* were lower in other seasons than in winter (pooled OR = 0.71; 95% CI = 0.65–0.77; I^2^ = 0.0%). AMR rates of *S*. *pneumoniae* to penicillins were also lower in other seasons than in winter (pooled OR = 0.68. 95% CI = 0.60–0.76. I^2^ = 14.4%) [3–5, 10, 11, 13] (Fig 2).

**Fig 3:**
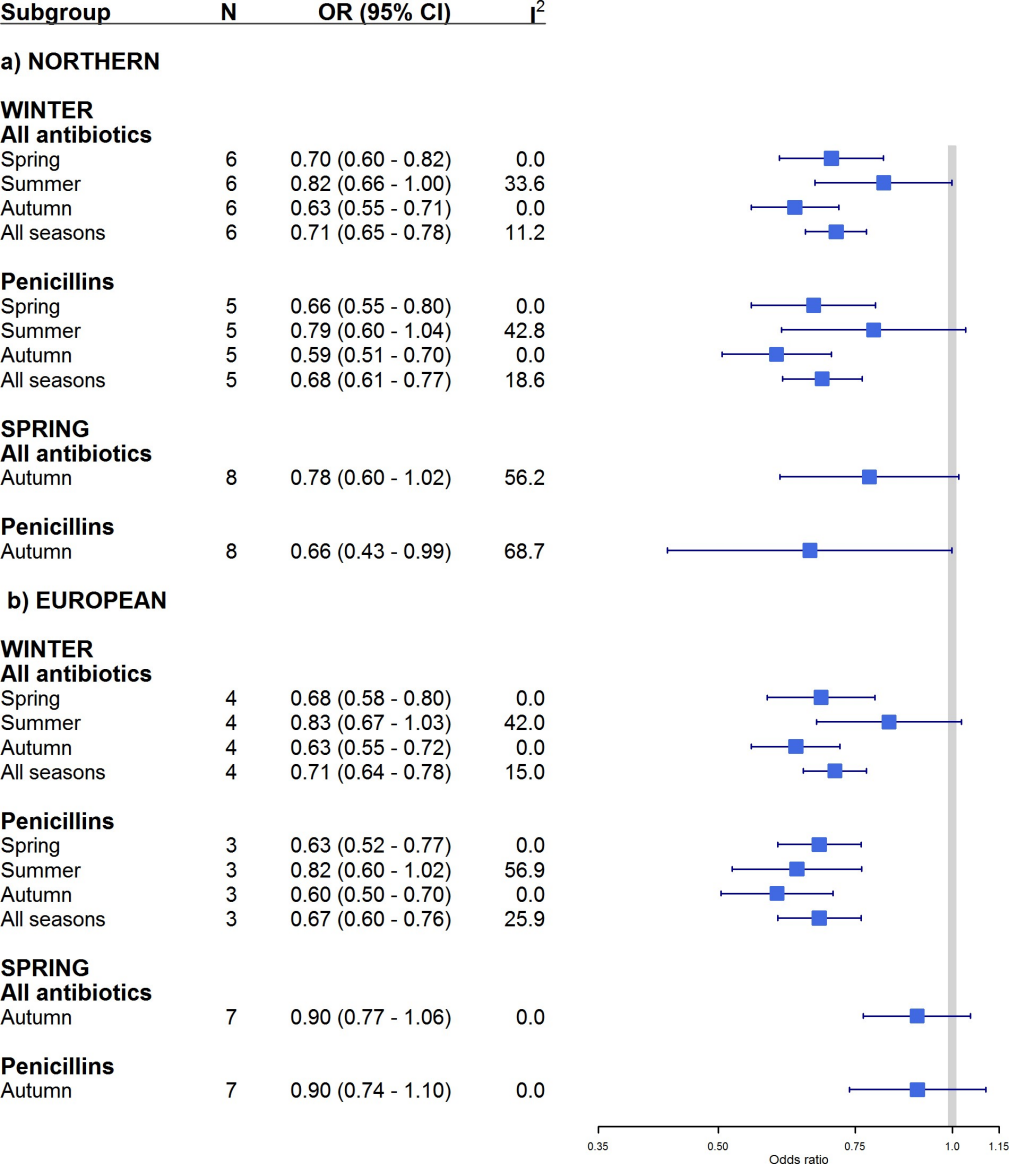
Forest plot of seasonality of antimicrobial resistance rates in *Streptococcus pneumoniae* by geographical region. Studies were stratified into two subgroups of antibiotics and estimates of effect are presented as pooled odds ratios (squares) with 95% confidence intervals (lateral lines of squares). For comparison, winter and spring were the reference groups, thus equal to one. Solid vertical line limits no difference between the two groups. I^2^ refers to percentage of heterogeneity among studies. The “All antibiotics” group include penicillins, cephalosporins, and Multi-drug resistance. Northern refers to studies in the Northern hemisphere; European refers to studies done in Europe.

A sensitivity analysis was done only by excluding the two studies where the denominador was imputed, and the seasonal variation of AMR rates remained the same (S1 Fig). In additional analyses, we did not find differences in AMR rates of *S*. *pneumoniae* between spring and autumn independently of geographical region and antibiotic class (Fig 3). The description of the study weights for each meta-analysis are present in the supporting information S4 Table.

We identified relatively low heterogeneity (I^2^<50%) among included studies, except in comparisons between autumn and spring (I^2^>60%) (Figs 2 and 3). Based on observation of funnel plots, there was no evidence of publication bias (S2 Fig). However, we identified larger effect sizes of small-studies (Egger P<0.05) when comparing between summer and winter (S5 Table).

## Discussion

In this study we describe the seasonality of AMR rates in two respiratory bacteria *S*. *pneumoniae* and *H*. *influenzae*. Few studies have been done in *H*. *influenzae* and no meta-analysis was performed, but according to the available evidence AMR rates tended to be higher in winter than summer. We found a consistent winter-peak of AMR rates in *S*. *pneumoniae* for penicillin and other antibiotics (i.e. cephalosporins, macrolides) independently of the geographical region.

The seasonal variation of AMR rates could results from a dynamic interaction of factors closely related with the seasonality of the infectious diseases such as higher antibiotic consumption [9, 18, 26], an increase of resistant strains during the peak of the infection [4, 12]. A 3-year population-based study in the USA, showed that the incidence rate of pneumococcal disease varied from 10 cases per 100.000 population in summer to 35–70 cases per 100.000 population in winter [27]. It was suggested that differences in host susceptibility, environmental factors, population behavior, and interactions among pathogens are determinants for seasonal incidence of pneumococcal infections [27–29]. For example, the winter-peak of incidence rates of pneumonococcal infections was associated with photoperiod variation and inversely correlated with temperature (i.e. increased number of hours of darkness with cold temperatures)[27], which would results in a higher pathogen abundance in winter. Besides, it was shown significant correlation between the winter-peak of respiratory syncytial virus and penicillin-resistant *S*. *pneumoniae* peaking in the winter [29], and thus the potential interaction with respiratory virus may actively participate in the winter seasonal peak of AMR rates.

Furthermore, seasonal variation in outpatient antibiotic use has been reported in Europe and the United States. The total antibiotic use increase between 24% to 38% in the winter compared with summer [9, 18, 26], mostly due to the increase of use of penicillins cephalosporins, and macrolides, which are broadly used to treat respiratory infections [30]. Other studies reported also high use amoxicillin, amoxicillin-clavulanic acid, and quinolones in winter to treat upper respiratory infections [15]. This increase of antibiotic use in winter could lead to periods of multidrug-resistant isoaltes peaking. Moreover, the high antibiotic prescription rates in the winter have been found to be innapropiate, as also viral respiratory infections ocurr at higher rates during winter [9, 31, 32]. In a population-based study in the United States, it was shownthat acute respiratory infections (e.g. sinusitis, otitits media, pnuemoniae) account for 211 prescriptions per 1000 person per year, of which 111 prescription were estimated to be inappropriate [32]. This overuse probably increases the selection of resistant bacteria, and harbors the risk of spread in the community due to indoor activities during winter [28].

Although we did not analyze the seasonal variation of outpatient antibiotic use, it was previously observed that a reduction of the total amount of antibiotic use in summer correlated with an overall reduction of MDR and single-drug resistance in *S*. *pneumoniae* isolates in the same season [14]. Similarly, a significant reduction of penicillin-resistant *S*. *pneumoniae* from 53% to 7% was found after a marked reduction of antibiotic use over a 4-month period [25]. Apparently, AMR impose high fitness cost on *S*. *pneumoniae* isolates reducing the transmissibility after antibiotic pressure is reduced [14]. Therefore, it may be expected that the reduction of antibiotic use in winter and spring will reduce AMR in the community in the same season. However, future studies are necessary to understand the seasonal and long-term impact of decreasing antibiotic use on resistance selection.

We did not find seasonal differences in rates in *S*. *pneumoniae* according to geographical region, despite that some studies show differences in choice of antibiotic class at various levels of the healthcare system (e.g. primary and secondary care) across regions, and in the structure of the pharmaceutical market across countries [9, 18]. Furthermore, within a country, socio-cultural determinants and educational level can influence the patient demands and the proclivity of medical doctors for prescribing antibiotics [33], leading to regional differences in prescription patterns. Regional differences in outpatients antibiotic use were shown in Europe; the Southern and Eastern regions showed a mean winter increase of antibiotic use of about 35%, whereas in Northern regions the use was 25% less in winter, compared to summer [18, 26].

We could not perfom a meta-analysis in *H*. *influenzae*; however, it can be argued that the seasonality of AMR rates will be similar to that in *S*. *pneumoniae*. These two bacteria are normally part of the nasopharyngeal flora and, the incidence of *H*. *influenzae* infections occurs at higher rates in winter and spring compared with summer and autumn [17].

### Clinical implications

In clinical settings, and from a public health perspective, the present review suggests a need to adress the seasonality of infectious diseases and its subsequent antibiotic use in winter. The high use of certain types of antibiotics in winter warrants attention because of the possible co-selection of resistance and further spread of MDR in clinically important bacteria. Thus, several efforts may be implemented to optimize the use of antibiotics in a specific season.

First, increasing collaboration among physicians prescribing antibiotics may be fundamental to promote prudent antibiotic use by enhancing stewardship programs in primary care and hospital settings. According to the United States Center for Disease Control and Prevention (CDC), appropiate antibiotic stewardship should be properly structured considering the core elements including commitment, implementation of polices and practices, tracking and reporting antibiotic use and resistance, and educational programs to patients and clinicians [34]. We believe that the seasonal variation of the incidence of infectious diseases should be part of such programs to reduce it impact in the seasonality of AMR rates in the community.

Second, implementing educational programs regarding appropriate antibiotic use for patients are necessary. Educational programs have proven to be effective in the reduction of outpatient antibiotic use; for instance, a population-based study in France showed that an intensive educational program helped to decrease the number of prescription and to change the dose/duration of the treatment leading to a reduction of penicillin-resistant *S*. *pneumoniae* carriage in children [35]. Such program should be carried out during the winter season for a higher impact.

Third, according to the WHO, the prevention strategies for infectious diseases are mainly based on vaccination, access to non-contaminated water, sanitation and hygiene in homes, school and health care facilities [36]. This needs to be addressed at the start of the winter season when people are crowding inside. Therefore, international support is necessary to implement programs and health campaings to increase the awareness of respiratory infections burden. Indeed, in Germany a health campaings implemented in autumn and winter increased vaccine uptake in elderly people and resulted ina decrease in number of pneumonias [37].

Finally, new strategies are needed at the start of the winter season to reduce the use of antibiotics among such as, implementation of treatment guidelines and appropriate use of diagnostic tests. For instance, in Turkey, a combination of respiratoy viral panel (Multiplex PCR panel) and rapid detection of streptococcus antigen limited the use of antibioticis in clinical settings [31]. In primary care, the implementation of point-of-care testing (POCT) of C-reactive protein (CRP) and traning to improve communication skills among general practitioners were effective to optimize antibiotic use use [38]. Recently, in the United Kindom, POCT effectiveness was proved by observing a shift in the prescription pattern among general practitioners to less prescribing of antibiotics [39].

### Strengths and limitations

To our knowledge, this is the first comprehensive systematic review and meta-analysis addressing the seasonality of AMR rates in respiraty bacteria and the possible factors underlying this pattern. However, some limitations need to be acknowledged. Firstly, we could not systematically examine the direct effect of the seasonal variation of antibiotic use and seasonality of infections diseases, because these factors were not examined in most included studies.

Secondly, the meta-analysis we have included two studies assuming equally distributed sampling throughout the study period. This assumption could not be always true, it could be also expected that more isolates are taken in peak-periods of the infection, thus more likely to have peaks in resistance due to higher exposure to antibiotics. However, sensitivity analysis was done comparing results with and without these studies and the seasonality of AMR rates in *S*. *pneumoniae* remained the same.

Finally, we cannot completely rule out publication bias and its influence on our meta-analysis, because most of the studies were performed in Europe. We could not included six studies in our meta-analysis, because it was not possible to categorize the four seasons. Thus, the potential variation that could occur in countries with a different climate conditions, seasonal incidence of infections and antibiotic use could be underrepresented.

## Conclusion

In this comprehensive systematic review, we found a consistent winter-peak seasonal variation of AMR rates in *S*. *pneumoniae* to penicillin and to all antibiotics, independently of geographical region. Due to the few available studies, we could not perform a quantitative analysis of seasonality of AMR in *H*. *influenzae*. The seasonality of AMR rates could result from the seasonality of infectious diseases and the accompanying antibiotic use. Future studies are required to better understand the factors underlying the seasonality of AMR rates such as the seasonal variation of antibiotic use and its temporal association with antimicrobial resistance in clinically important bacteria.

## Supporting information

S1 Table PRISMA checklist. (DOCX)

S1 Text Databases search strategy and terms. (DOCX)

S2 Text Modified version of the Newcastle-Ottawa Scale (NOS) for cross-sectional studies. (DOCX)

S2 Table Quality assessment of included studies (n = 13). (DOCX)

S3 Table Description of studies describing seasonality of antimicrobial resistance rates in respiratory bacteria. (DOCX)

S4 Table Description of study weights in each meta-analysis. (DOCX)

S5 Table Assessment of publication bias. Egger´s test.
(DOCX)

S1 Fig Sensitivity analysis forest-plot. (DOCX)

S2 Fig Funnel plots. (DOCX)
