# Supplementary material for: Seasonality of antimicrobial resistance rates in respiratory bacteria: A systematic review and meta-analysis
Source: PLoS One. 2019 Aug 15;14(8):e0221133. doi: 10.1371/journal.pone.0221133 (PMC6695168; doi:10.1371/journal.pone.0221133)
Supplement: S1 Text — (DOCX) [file pone.0221133.s002.docx]

# S1 Text. Databases search strategy and terms

**First search including all bacteria.**

**Embase.com**

('antibiotic sensitivity'/exp OR 'antibiotic agent'/exp/dd_ad OR 'penicillin resistance'/de OR (('drug resistance'/de OR 'drug sensitivity'/de) AND 'antiinfective agent'/exp) OR 'multidrug resistance'/exp OR (((antibiotic* OR antibacter* OR anti-biotic* OR antiinfect* OR anti-infect* OR anti-bacter* OR antimicrob* OR anti-microb* OR penicillin* OR aminoglycosid* OR lactam* OR cephalosporin* OR fenicole* OR fluorochinono* OR macrolide* OR trimethoprim* OR tetracyclin* OR sulphamide* OR abyssomicin* OR acetomycin* OR actinorhodine* OR aditoprim* OR agglomerin* OR alafosfalin* OR aldecalmycin* OR alisamycin* OR allicin* OR ambruticin* OR ansamitocin* OR ansamycin* OR aplasmomycin* OR aristeromycin* OR asukamycin* OR atpenin* OR auricularum* OR aurograb* OR avilamycin* OR bafilomycin* OR baliz* OR baquiloprim* OR beroline* OR betafectin* OR betamipron* OR boromycin* OR borrelidin* OR brilacidin* OR butalactin* OR cadazolid* OR calcimycin* OR carbadox* OR carbazomycin* OR chloramphenicol* OR ciadox* OR cinoquidox* OR citrinin* OR concanamycin* OR coumamycin* OR cryptosporin* OR cycloheximide* OR dalfopristin* OR dealanylalahopcin* OR dioxidine* OR echinomycin* OR edeine* OR efepristin* OR emimycin* OR endusamycin* OR eperezolid* OR epetraborole* OR epiderstatin* OR epiroprim* OR ethylhydrocupreine* OR evernimicin* OR everninomicin* OR flopristin* OR fosmidomycin* OR furaquinocin* OR furazidin* OR furazolium* OR fusafungine* OR fusidate-sodium* OR fusidic-acid* OR gepotidacin* OR grisein* OR hatomamicin* OR hedamycin* OR heliomycin* OR hidamicin* OR hymeglusin* OR iclaprim* OR ikarugamycin* OR inostamycin* OR kalafungin* OR kelfiprim* OR kidamycin* OR kinamycin* OR kinamycin* OR lactacystin* OR lactivicin* OR laidlomycin* OR lanopepden* OR lasalocid* OR lavanducyanin* OR lenoremycin* OR linezolid* OR linopristin* OR lonomycin* OR lotilibcin* OR lydicamycin* OR lysocellin* OR macrolide* OR malyngolide* OR manumycin* OR methylenomycin* OR mikamycin* OR monensin* OR monensin* OR mureidomycin* OR mycolog* OR myxothiazol* OR narasin* OR negamycin* OR nybomycin* OR olaquindox* OR paldimycin* OR patulin* OR pentalenolactone* OR platensimycin* OR pluramycin* OR polyactin* OR polyfungin* OR posizolid* OR pristinamycin* OR prothracarcin* OR pseudomonic-acid* OR pyrroxamycin* OR quinomycin* OR quinupristin* OR radezolid* OR radicicol* OR ranbezolid* OR simaomicin* OR simocyclinone* OR spectinomycin* OR squalamine* OR streptogramin* OR streptovitacin* OR tedizolid* OR terdecamycin* OR tetracycline* OR tetronasin* OR tetronomycin* OR tetroxoprim* OR thiolactomycin* OR tibezonium-iodide* OR tizoxanide* OR toyocamycin* OR toyocamycin* OR trichostatic-acid* OR trichostatin* OR trimethoprim* OR trimethoprim* OR triostin* OR trospectomycin* OR tuftsin* OR tuftsin* OR tutofusin* OR urdamycin* OR validamycin* OR vernamycin* OR virginiae-butanolide* OR virginiamycin* OR volpristin* OR zibrofusidic-acid* OR zorbamycin* OR fluoroquinolon* OR quinolon* OR multidrug* OR multi-drug* OR methicillin* OR ticarcillin* OR ampicillin* OR ciprofloxacin* OR cefprozil* OR cefaclor* OR amoxicillin* OR streptomycin*) NEAR/10 (sensitiv* OR susceptib*)) OR resistan*):ab,ti) AND ('Campylobacter'/exp OR 'campylobacteriosis'/exp OR 'Salmonella'/exp OR 'salmonellosis'/de OR 'Escherichia coli'/exp OR 'Escherichia coli infection'/exp OR 'Streptococcus pneumoniae'/exp OR 'pneumococcal infection'/exp OR 'Haemophilus influenzae'/exp OR 'Haemophilus infection'/exp OR 'bacterial meningitis'/exp OR 'bacterial pneumonia'/exp OR 'otitis media'/de OR 'acute otitis media'/de OR (Campylobacter* OR Salmonell* OR 'Escherichia coli' OR e-coli OR (Streptococc* NEAR/3 pneumon*) OR (Haemoph* NEAR/3 (influen* OR meningit*)) OR ((respirator* OR food-born* OR foodborn*) NEAR/3 pathogen*) OR ((enteric* OR typhoid*) NEAR/3 fever*) OR pneumococc* OR (bacteri* NEAR/3 meningit*) OR (otitis NEAR/3 (media OR infect*)) OR ((Communit* OR bacter*) NEAR/3 pneumonia*)):ab,ti) AND ('temporal analysis'/exp OR 'meteorological phenomena'/exp OR ((time NEAR/3 series*) OR season* OR Autumn* OR spring OR summer* OR winter* OR meteorolog* OR weather* OR climate* OR ((temporal* OR month* ) NEAR/6 (variat* OR higher* OR lower* OR compar* OR divers* OR risk OR fluctuat* OR peak OR pattern* OR dynamic* OR trend* OR monitor* OR decline* OR decrease* OR increase* OR incline* OR change* OR associat*)) OR (throughout NEAR/3 year) ):ab,ti)

**Medline Ovid**

(exp "Drug Resistance, Microbial"/ OR (("Drug Resistance"/ ) AND exp "Anti-Infective Agents"/) OR "Drug Resistance, Multiple"/ OR (((antibiotic* OR antibacter* OR anti-biotic* OR antiinfect* OR anti-infect* OR anti-bacter* OR antimicrob* OR anti-microb* OR penicillin* OR aminoglycosid* OR lactam* OR cephalosporin* OR fenicole* OR fluorochinono* OR macrolide* OR trimethoprim* OR tetracyclin* OR sulphamide* OR abyssomicin* OR acetomycin* OR actinorhodine* OR aditoprim* OR agglomerin* OR alafosfalin* OR aldecalmycin* OR alisamycin* OR allicin* OR ambruticin* OR ansamitocin* OR ansamycin* OR aplasmomycin* OR aristeromycin* OR asukamycin* OR atpenin* OR auricularum* OR aurograb* OR avilamycin* OR bafilomycin* OR baliz* OR baquiloprim* OR beroline* OR betafectin* OR betamipron* OR boromycin* OR borrelidin* OR brilacidin* OR butalactin* OR cadazolid* OR calcimycin* OR carbadox* OR carbazomycin* OR chloramphenicol* OR ciadox* OR cinoquidox* OR citrinin* OR concanamycin* OR coumamycin* OR cryptosporin* OR cycloheximide* OR dalfopristin* OR dealanylalahopcin* OR dioxidine* OR echinomycin* OR edeine* OR efepristin* OR emimycin* OR endusamycin* OR eperezolid* OR epetraborole* OR epiderstatin* OR epiroprim* OR ethylhydrocupreine* OR evernimicin* OR everninomicin* OR flopristin* OR fosmidomycin* OR furaquinocin* OR furazidin* OR furazolium* OR fusafungine* OR fusidate-sodium* OR fusidic-acid* OR gepotidacin* OR grisein* OR hatomamicin* OR hedamycin* OR heliomycin* OR hidamicin* OR hymeglusin* OR iclaprim* OR ikarugamycin* OR inostamycin* OR kalafungin* OR kelfiprim* OR kidamycin* OR kinamycin* OR kinamycin* OR lactacystin* OR lactivicin* OR laidlomycin* OR lanopepden* OR lasalocid* OR lavanducyanin* OR lenoremycin* OR linezolid* OR linopristin* OR lonomycin* OR lotilibcin* OR lydicamycin* OR lysocellin* OR macrolide* OR malyngolide* OR manumycin* OR methylenomycin* OR mikamycin* OR monensin* OR monensin* OR mureidomycin* OR mycolog* OR myxothiazol* OR narasin* OR negamycin* OR nybomycin* OR olaquindox* OR paldimycin* OR patulin* OR pentalenolactone* OR platensimycin* OR pluramycin* OR polyactin* OR polyfungin* OR posizolid* OR pristinamycin* OR prothracarcin* OR pseudomonic-acid* OR pyrroxamycin* OR quinomycin* OR quinupristin* OR radezolid* OR radicicol* OR ranbezolid* OR simaomicin* OR simocyclinone* OR spectinomycin* OR squalamine* OR streptogramin* OR streptovitacin* OR tedizolid* OR terdecamycin* OR tetracycline* OR tetronasin* OR tetronomycin* OR tetroxoprim* OR thiolactomycin* OR tibezonium-iodide* OR tizoxanide* OR toyocamycin* OR toyocamycin* OR trichostatic-acid* OR trichostatin* OR trimethoprim* OR trimethoprim* OR triostin* OR trospectomycin* OR tuftsin* OR tuftsin* OR tutofusin* OR urdamycin* OR validamycin* OR vernamycin* OR virginiae-butanolide* OR virginiamycin* OR volpristin* OR zibrofusidic-acid* OR zorbamycin* OR fluoroquinolon* OR quinolon* OR multidrug* OR multi-drug* OR methicillin* OR ticarcillin* OR ampicillin* OR ciprofloxacin* OR cefprozil* OR cefaclor* OR amoxicillin* OR streptomycin*) ADJ10 (sensitiv* OR susceptib*)) OR resistan*).ab,ti.) AND (exp "Campylobacter"/ OR exp "Campylobacter Infections"/ OR exp "Salmonella"/ OR exp "Salmonella Infections"/ OR exp "Escherichia coli"/ OR exp "Escherichia coli Infections"/ OR exp "Streptococcus pneumoniae"/ OR exp "Pneumococcal Infections"/ OR exp "Haemophilus influenzae"/ OR exp "Haemophilus Infections"/ OR exp "Meningitis, Bacterial"/ OR exp "Pneumonia, Bacterial"/ OR "otitis media"/ OR (Campylobacter* OR Salmonell* OR "Escherichia coli" OR e-coli OR (Streptococc* ADJ3 pneumon*) OR (Haemoph* ADJ3 (influen* OR meningit*)) OR ((respirator* OR food-born* OR foodborn*) ADJ3 pathogen*) OR ((enteric* OR typhoid*) ADJ3 fever*) OR pneumococc* OR (bacteri* ADJ3 meningit*) OR (otitis ADJ3 (media OR infect*)) OR ((Communit* OR bacter*) ADJ3 pneumonia*)).ab,ti.) AND ("Climate"/ OR Seasons/ OR Weather/ OR ((time ADJ3 series*) OR season* OR Autumn* OR spring OR summer* OR winter* OR meteorolog* OR weather* OR climate* OR ((temporal* OR month* ) ADJ6 (variat* OR higher* OR lower* OR compar* OR divers* OR risk OR fluctuat* OR peak OR pattern* OR dynamic* OR trend* OR monitor* OR decline* OR decrease* OR increase* OR incline* OR change* OR associat*)) OR (throughout ADJ3 year) ).ab,ti.)

**Cochrane**

((((antibiotic* OR antibacter* OR anti-biotic* OR antiinfect* OR anti-infect* OR anti-bacter* OR antimicrob* OR anti-microb* OR penicillin* OR aminoglycosid* OR lactam* OR cephalosporin* OR fenicole* OR fluorochinono* OR macrolide* OR trimethoprim* OR tetracyclin* OR sulphamide* OR abyssomicin* OR acetomycin* OR actinorhodine* OR aditoprim* OR agglomerin* OR alafosfalin* OR aldecalmycin* OR alisamycin* OR allicin* OR ambruticin* OR ansamitocin* OR ansamycin* OR aplasmomycin* OR aristeromycin* OR asukamycin* OR atpenin* OR auricularum* OR aurograb* OR avilamycin* OR bafilomycin* OR baliz* OR baquiloprim* OR beroline* OR betafectin* OR betamipron* OR boromycin* OR borrelidin* OR brilacidin* OR butalactin* OR cadazolid* OR calcimycin* OR carbadox* OR carbazomycin* OR chloramphenicol* OR ciadox* OR cinoquidox* OR citrinin* OR concanamycin* OR coumamycin* OR cryptosporin* OR cycloheximide* OR dalfopristin* OR dealanylalahopcin* OR dioxidine* OR echinomycin* OR edeine* OR efepristin* OR emimycin* OR endusamycin* OR eperezolid* OR epetraborole* OR epiderstatin* OR epiroprim* OR ethylhydrocupreine* OR evernimicin* OR everninomicin* OR flopristin* OR fosmidomycin* OR furaquinocin* OR furazidin* OR furazolium* OR fusafungine* OR fusidate-sodium* OR fusidic-acid* OR gepotidacin* OR grisein* OR hatomamicin* OR hedamycin* OR heliomycin* OR hidamicin* OR hymeglusin* OR iclaprim* OR ikarugamycin* OR inostamycin* OR kalafungin* OR kelfiprim* OR kidamycin* OR kinamycin* OR kinamycin* OR lactacystin* OR lactivicin* OR laidlomycin* OR lanopepden* OR lasalocid* OR lavanducyanin* OR lenoremycin* OR linezolid* OR linopristin* OR lonomycin* OR lotilibcin* OR lydicamycin* OR lysocellin* OR macrolide* OR malyngolide* OR manumycin* OR methylenomycin* OR mikamycin* OR monensin* OR monensin* OR mureidomycin* OR mycolog* OR myxothiazol* OR narasin* OR negamycin* OR nybomycin* OR olaquindox* OR paldimycin* OR patulin* OR pentalenolactone* OR platensimycin* OR pluramycin* OR polyactin* OR polyfungin* OR posizolid* OR pristinamycin* OR prothracarcin* OR pseudomonic-acid* OR pyrroxamycin* OR quinomycin* OR quinupristin* OR radezolid* OR radicicol* OR ranbezolid* OR simaomicin* OR simocyclinone* OR spectinomycin* OR squalamine* OR streptogramin* OR streptovitacin* OR tedizolid* OR terdecamycin* OR tetracycline* OR tetronasin* OR tetronomycin* OR tetroxoprim* OR thiolactomycin* OR tibezonium-iodide* OR tizoxanide* OR toyocamycin* OR toyocamycin* OR trichostatic-acid* OR trichostatin* OR trimethoprim* OR trimethoprim* OR triostin* OR trospectomycin* OR tuftsin* OR tuftsin* OR tutofusin* OR urdamycin* OR validamycin* OR vernamycin* OR virginiae-butanolide* OR virginiamycin* OR volpristin* OR zibrofusidic-acid* OR zorbamycin* OR fluoroquinolon* OR quinolon* OR multidrug* OR multi-drug* OR methicillin* OR ticarcillin* OR ampicillin* OR ciprofloxacin* OR cefprozil* OR cefaclor* OR amoxicillin* OR streptomycin*) NEAR/10 (sensitiv* OR susceptib*)) OR resistan*):ab,ti) AND ((Campylobacter* OR Salmonell* OR Escherichia-coli OR e-coli OR (Streptococc* NEAR/3 pneumon*) OR (Haemoph* NEAR/3 (influen* OR meningit*)) OR ((respirator* OR food-born* OR foodborn*) NEAR/3 pathogen*) OR ((enteric* OR typhoid*) NEAR/3 fever*) OR pneumococc* OR (bacteri* NEAR/3 meningit*) OR (otitis NEAR/3 (media OR infect*)) OR ((Communit* OR bacter*) NEAR/3 pneumonia*)):ab,ti) AND (((time NEAR/3 series*) OR season* OR Autumn* OR spring OR summer* OR winter* OR meteorolog* OR weather* OR climate* OR ((temporal* OR month* ) NEAR/6 (variat* OR higher* OR lower* OR compar* OR divers* OR risk OR fluctuat* OR peak OR pattern* OR dynamic* OR trend* OR monitor* OR decline* OR decrease* OR increase* OR incline* OR change* OR associat*)) OR (throughout NEAR/3 year) OR periodicity):ab,ti)

**Web of science**

TS=(((((antibiotic* OR antibacter* OR anti-biotic* OR antiinfect* OR anti-infect* OR anti-bacter* OR antimicrob* OR anti-microb* OR penicillin* OR aminoglycosid* OR lactam* OR cephalosporin* OR fenicole* OR fluorochinono* OR macrolide* OR trimethoprim* OR tetracyclin* OR sulphamide* OR abyssomicin* OR acetomycin* OR actinorhodine* OR aditoprim* OR agglomerin* OR alafosfalin* OR aldecalmycin* OR alisamycin* OR allicin* OR ambruticin* OR ansamitocin* OR ansamycin* OR aplasmomycin* OR aristeromycin* OR asukamycin* OR atpenin* OR auricularum* OR aurograb* OR avilamycin* OR bafilomycin* OR baliz* OR baquiloprim* OR beroline* OR betafectin* OR betamipron* OR boromycin* OR borrelidin* OR brilacidin* OR butalactin* OR cadazolid* OR calcimycin* OR carbadox* OR carbazomycin* OR chloramphenicol* OR ciadox* OR cinoquidox* OR citrinin* OR concanamycin* OR coumamycin* OR cryptosporin* OR cycloheximide* OR dalfopristin* OR dealanylalahopcin* OR dioxidine* OR echinomycin* OR edeine* OR efepristin* OR emimycin* OR endusamycin* OR eperezolid* OR epetraborole* OR epiderstatin* OR epiroprim* OR ethylhydrocupreine* OR evernimicin* OR everninomicin* OR flopristin* OR fosmidomycin* OR furaquinocin* OR furazidin* OR furazolium* OR fusafungine* OR fusidate-sodium* OR fusidic-acid* OR gepotidacin* OR grisein* OR hatomamicin* OR hedamycin* OR heliomycin* OR hidamicin* OR hymeglusin* OR iclaprim* OR ikarugamycin* OR inostamycin* OR kalafungin* OR kelfiprim* OR kidamycin* OR kinamycin* OR kinamycin* OR lactacystin* OR lactivicin* OR laidlomycin* OR lanopepden* OR lasalocid* OR lavanducyanin* OR lenoremycin* OR linezolid* OR linopristin* OR lonomycin* OR lotilibcin* OR lydicamycin* OR lysocellin* OR macrolide* OR malyngolide* OR manumycin* OR methylenomycin* OR mikamycin* OR monensin* OR monensin* OR mureidomycin* OR mycolog* OR myxothiazol* OR narasin* OR negamycin* OR nybomycin* OR olaquindox* OR paldimycin* OR patulin* OR pentalenolactone* OR platensimycin* OR pluramycin* OR polyactin* OR polyfungin* OR posizolid* OR pristinamycin* OR prothracarcin* OR pseudomonic-acid* OR pyrroxamycin* OR quinomycin* OR quinupristin* OR radezolid* OR radicicol* OR ranbezolid* OR simaomicin* OR simocyclinone* OR spectinomycin* OR squalamine* OR streptogramin* OR streptovitacin* OR tedizolid* OR terdecamycin* OR tetracycline* OR tetronasin* OR tetronomycin* OR tetroxoprim* OR thiolactomycin* OR tibezonium-iodide* OR tizoxanide* OR toyocamycin* OR toyocamycin* OR trichostatic-acid* OR trichostatin* OR trimethoprim* OR trimethoprim* OR triostin* OR trospectomycin* OR tuftsin* OR tuftsin* OR tutofusin* OR urdamycin* OR validamycin* OR vernamycin* OR virginiae-butanolide* OR virginiamycin* OR volpristin* OR zibrofusidic-acid* OR zorbamycin* OR fluoroquinolon* OR quinolon* OR multidrug* OR multi-drug* OR methicillin* OR ticarcillin* OR ampicillin* OR ciprofloxacin* OR cefprozil* OR cefaclor* OR amoxicillin* OR streptomycin*) NEAR/9 (sensitiv* OR susceptib*)) OR resistan*)) AND ((Campylobacter* OR Salmonell* OR "Escherichia coli" OR e-coli OR (Streptococc* NEAR/2 pneumon*) OR (Haemoph* NEAR/2 (influen* OR meningit*)) OR ((respirator* OR food-born* OR foodborn*) NEAR/2 pathogen*) OR ((enteric* OR typhoid*) NEAR/2 fever*) OR pneumococc* OR (bacteri* NEAR/2 meningit*) OR (otitis NEAR/2 (media OR infect*)) OR ((Communit* OR bacter*) NEAR/2 pneumonia*))) AND (((time NEAR/2 series*) OR season* OR Autumn* OR spring OR summer* OR winter* OR meteorolog* OR weather* OR climate* OR ((temporal* OR month* ) NEAR/5 (variat* OR higher* OR lower* OR compar* OR divers* OR risk OR fluctuat* OR peak OR pattern* OR dynamic* OR trend* OR monitor* OR decline* OR decrease* OR increase* OR incline* OR change* OR associat*)) OR (throughout NEAR/2 year) OR "periodicity"))).

**Biosis Ovid** – not updated, since this database is not available for us (anymore), used the file of 26/09/2017

((((antibiotic* OR antibacter* OR anti-biotic* OR antiinfect* OR anti-infect* OR anti-bacter* OR antimicrob* OR anti-microb* OR penicillin* OR aminoglycosid* OR lactam* OR cephalosporin* OR fenicole* OR fluorochinono* OR macrolide* OR trimethoprim* OR tetracyclin* OR sulphamide* OR abyssomicin* OR acetomycin* OR actinorhodine* OR aditoprim* OR agglomerin* OR alafosfalin* OR aldecalmycin* OR alisamycin* OR allicin* OR ambruticin* OR ansamitocin* OR ansamycin* OR aplasmomycin* OR aristeromycin* OR asukamycin* OR atpenin* OR auricularum* OR aurograb* OR avilamycin* OR bafilomycin* OR baliz* OR baquiloprim* OR beroline* OR betafectin* OR betamipron* OR boromycin* OR borrelidin* OR brilacidin* OR butalactin* OR cadazolid* OR calcimycin* OR carbadox* OR carbazomycin* OR chloramphenicol* OR ciadox* OR cinoquidox* OR citrinin* OR concanamycin* OR coumamycin* OR cryptosporin* OR cycloheximide* OR dalfopristin* OR dealanylalahopcin* OR dioxidine* OR echinomycin* OR edeine* OR efepristin* OR emimycin* OR endusamycin* OR eperezolid* OR epetraborole* OR epiderstatin* OR epiroprim* OR ethylhydrocupreine* OR evernimicin* OR everninomicin* OR flopristin* OR fosmidomycin* OR furaquinocin* OR furazidin* OR furazolium* OR fusafungine* OR fusidate-sodium* OR fusidic-acid* OR gepotidacin* OR grisein* OR hatomamicin* OR hedamycin* OR heliomycin* OR hidamicin* OR hymeglusin* OR iclaprim* OR ikarugamycin* OR inostamycin* OR kalafungin* OR kelfiprim* OR kidamycin* OR kinamycin* OR kinamycin* OR lactacystin* OR lactivicin* OR laidlomycin* OR lanopepden* OR lasalocid* OR lavanducyanin* OR lenoremycin* OR linezolid* OR linopristin* OR lonomycin* OR lotilibcin* OR lydicamycin* OR lysocellin* OR macrolide* OR malyngolide* OR manumycin* OR methylenomycin* OR mikamycin* OR monensin* OR monensin* OR mureidomycin* OR mycolog* OR myxothiazol* OR narasin* OR negamycin* OR nybomycin* OR olaquindox* OR paldimycin* OR patulin* OR pentalenolactone* OR platensimycin* OR pluramycin* OR polyactin* OR polyfungin* OR posizolid* OR pristinamycin* OR prothracarcin* OR pseudomonic-acid* OR pyrroxamycin* OR quinomycin* OR quinupristin* OR radezolid* OR radicicol* OR ranbezolid* OR simaomicin* OR simocyclinone* OR spectinomycin* OR squalamine* OR streptogramin* OR streptovitacin* OR tedizolid* OR terdecamycin* OR tetracycline* OR tetronasin* OR tetronomycin* OR tetroxoprim* OR thiolactomycin* OR tibezonium-iodide* OR tizoxanide* OR toyocamycin* OR toyocamycin* OR trichostatic-acid* OR trichostatin* OR trimethoprim* OR trimethoprim* OR triostin* OR trospectomycin* OR tuftsin* OR tuftsin* OR tutofusin* OR urdamycin* OR validamycin* OR vernamycin* OR virginiae-butanolide* OR virginiamycin* OR volpristin* OR zibrofusidic-acid* OR zorbamycin* OR fluoroquinolon* OR quinolon* OR multidrug* OR multi-drug* OR methicillin* OR ticarcillin* OR ampicillin* OR ciprofloxacin* OR cefprozil* OR cefaclor* OR amoxicillin* OR streptomycin*) ADJ10 (sensitiv* OR susceptib*)) OR resistan*).ab,ti.) AND ((Campylobacter* OR Salmonell* OR "Escherichia coli" OR e-coli OR (Streptococc* ADJ3 pneumon*) OR (Haemoph* ADJ3 (influen* OR meningit*)) OR ((respirator* OR food-born* OR foodborn*) ADJ3 pathogen*) OR ((enteric* OR typhoid*) ADJ3 fever*) OR pneumococc* OR (bacteri* ADJ3 meningit*) OR (otitis ADJ3 (media OR infect*)) OR ((Communit* OR bacter*) ADJ3 pneumonia*)).ab,ti.) AND (" Climatology"/ OR ((time ADJ3 series*) OR season* OR Autumn* OR spring OR summer* OR winter* OR meteorolog* OR weather* OR climate* OR ((temporal* OR month* ) ADJ6 (variat* OR higher* OR lower* OR compar* OR divers* OR risk OR fluctuat* OR peak OR pattern* OR dynamic* OR trend* OR monitor* OR decline* OR decrease* OR increase* OR incline* OR change* OR associat*)) OR (throughout ADJ3 year) ).ab,ti.)

**Google scholar**

"antibiotic|antibacterial|biotic sensitivity|susceptibility|resistance" Campylobacter|Salmonella|"Escherichia|e coli"|Streptococca|Haemophilae|"respiratory|foodborne pathogens" "time series"|season|seasonal|weather|climate|"temporal variation|fluctuation

**Update Jun 19^th^, only including respiratory bacteria.**

**Embase.com 1100**

('antibiotic sensitivity'/exp OR 'antibiotic agent'/exp/dd_ad OR 'penicillin resistance'/de OR (('drug resistance'/de OR 'drug sensitivity'/de) AND 'antiinfective agent'/exp) OR 'multidrug resistance'/exp OR (((antibiotic* OR antibacter* OR anti-biotic* OR antiinfect* OR anti-infect* OR anti-bacter* OR antimicrob* OR anti-microb* OR penicillin* OR aminoglycosid* OR lactam* OR cephalosporin* OR fenicole* OR fluorochinono* OR macrolide* OR trimethoprim* OR tetracyclin* OR sulphamide* OR abyssomicin* OR acetomycin* OR actinorhodine* OR aditoprim* OR agglomerin* OR alafosfalin* OR aldecalmycin* OR alisamycin* OR allicin* OR ambruticin* OR ansamitocin* OR ansamycin* OR aplasmomycin* OR aristeromycin* OR asukamycin* OR atpenin* OR auricularum* OR aurograb* OR avilamycin* OR bafilomycin* OR baliz* OR baquiloprim* OR beroline* OR betafectin* OR betamipron* OR boromycin* OR borrelidin* OR brilacidin* OR butalactin* OR cadazolid* OR calcimycin* OR carbadox* OR carbazomycin* OR chloramphenicol* OR ciadox* OR cinoquidox* OR citrinin* OR concanamycin* OR coumamycin* OR cryptosporin* OR cycloheximide* OR dalfopristin* OR dealanylalahopcin* OR dioxidine* OR echinomycin* OR edeine* OR efepristin* OR emimycin* OR endusamycin* OR eperezolid* OR epetraborole* OR epiderstatin* OR epiroprim* OR ethylhydrocupreine* OR evernimicin* OR everninomicin* OR flopristin* OR fosmidomycin* OR furaquinocin* OR furazidin* OR furazolium* OR fusafungine* OR fusidate-sodium* OR fusidic-acid* OR gepotidacin* OR grisein* OR hatomamicin* OR hedamycin* OR heliomycin* OR hidamicin* OR hymeglusin* OR iclaprim* OR ikarugamycin* OR inostamycin* OR kalafungin* OR kelfiprim* OR kidamycin* OR kinamycin* OR kinamycin* OR lactacystin* OR lactivicin* OR laidlomycin* OR lanopepden* OR lasalocid* OR lavanducyanin* OR lenoremycin* OR linezolid* OR linopristin* OR lonomycin* OR lotilibcin* OR lydicamycin* OR lysocellin* OR macrolide* OR malyngolide* OR manumycin* OR methylenomycin* OR mikamycin* OR monensin* OR monensin* OR mureidomycin* OR mycolog* OR myxothiazol* OR narasin* OR negamycin* OR nybomycin* OR olaquindox* OR paldimycin* OR patulin* OR pentalenolactone* OR platensimycin* OR pluramycin* OR polyactin* OR polyfungin* OR posizolid* OR pristinamycin* OR prothracarcin* OR pseudomonic-acid* OR pyrroxamycin* OR quinomycin* OR quinupristin* OR radezolid* OR radicicol* OR ranbezolid* OR simaomicin* OR simocyclinone* OR spectinomycin* OR squalamine* OR streptogramin* OR streptovitacin* OR tedizolid* OR terdecamycin* OR tetracycline* OR tetronasin* OR tetronomycin* OR tetroxoprim* OR thiolactomycin* OR tibezonium-iodide* OR tizoxanide* OR toyocamycin* OR toyocamycin* OR trichostatic-acid* OR trichostatin* OR trimethoprim* OR trimethoprim* OR triostin* OR trospectomycin* OR tuftsin* OR tuftsin* OR tutofusin* OR urdamycin* OR validamycin* OR vernamycin* OR virginiae-butanolide* OR virginiamycin* OR volpristin* OR zibrofusidic-acid* OR zorbamycin* OR fluoroquinolon* OR quinolon* OR multidrug* OR multi-drug* OR methicillin* OR ticarcillin* OR ampicillin* OR ciprofloxacin* OR cefprozil* OR cefaclor* OR amoxicillin* OR streptomycin*) NEAR/10 (sensitiv* OR susceptib*)) OR resistan*):ab,ti) AND ('Streptococcus pneumoniae'/exp OR 'pneumococcal infection'/exp OR 'Haemophilus influenzae'/exp OR 'Haemophilus infection'/exp OR 'bacterial meningitis'/exp OR 'bacterial pneumonia'/exp OR 'otitis media'/de OR 'acute otitis media'/de OR ((Streptococc* NEAR/3 pneumon*) OR (Haemoph* NEAR/3 (influen* OR meningit*)) OR ((respirator* OR food-born* OR foodborn*) NEAR/3 pathogen*) OR ((enteric* OR typhoid*) NEAR/3 fever*) OR pneumococc* OR (bacteri* NEAR/3 meningit*) OR (otitis NEAR/3 (media OR infect*)) OR ((Communit* OR bacter*) NEAR/3 pneumonia*)):ab,ti) AND ('temporal analysis'/exp OR 'meteorological phenomena'/exp OR ((time NEAR/3 series*) OR season* OR Autumn* OR spring OR summer* OR winter* OR meteorolog* OR weather* OR climate* OR ((temporal* OR month* ) NEAR/6 (variat* OR higher* OR lower* OR compar* OR divers* OR risk OR fluctuat* OR peak OR pattern* OR dynamic* OR trend* OR monitor* OR decline* OR decrease* OR increase* OR incline* OR change* OR associat*)) OR (throughout NEAR/3 year) ):ab,ti)

**Medline Ovid 766**

(exp "Drug Resistance, Microbial"/ OR (("Drug Resistance"/ ) AND exp "Anti-Infective Agents"/) OR "Drug Resistance, Multiple"/ OR (((antibiotic* OR antibacter* OR anti-biotic* OR antiinfect* OR anti-infect* OR anti-bacter* OR antimicrob* OR anti-microb* OR penicillin* OR aminoglycosid* OR lactam* OR cephalosporin* OR fenicole* OR fluorochinono* OR macrolide* OR trimethoprim* OR tetracyclin* OR sulphamide* OR abyssomicin* OR acetomycin* OR actinorhodine* OR aditoprim* OR agglomerin* OR alafosfalin* OR aldecalmycin* OR alisamycin* OR allicin* OR ambruticin* OR ansamitocin* OR ansamycin* OR aplasmomycin* OR aristeromycin* OR asukamycin* OR atpenin* OR auricularum* OR aurograb* OR avilamycin* OR bafilomycin* OR baliz* OR baquiloprim* OR beroline* OR betafectin* OR betamipron* OR boromycin* OR borrelidin* OR brilacidin* OR butalactin* OR cadazolid* OR calcimycin* OR carbadox* OR carbazomycin* OR chloramphenicol* OR ciadox* OR cinoquidox* OR citrinin* OR concanamycin* OR coumamycin* OR cryptosporin* OR cycloheximide* OR dalfopristin* OR dealanylalahopcin* OR dioxidine* OR echinomycin* OR edeine* OR efepristin* OR emimycin* OR endusamycin* OR eperezolid* OR epetraborole* OR epiderstatin* OR epiroprim* OR ethylhydrocupreine* OR evernimicin* OR everninomicin* OR flopristin* OR fosmidomycin* OR furaquinocin* OR furazidin* OR furazolium* OR fusafungine* OR fusidate-sodium* OR fusidic-acid* OR gepotidacin* OR grisein* OR hatomamicin* OR hedamycin* OR heliomycin* OR hidamicin* OR hymeglusin* OR iclaprim* OR ikarugamycin* OR inostamycin* OR kalafungin* OR kelfiprim* OR kidamycin* OR kinamycin* OR kinamycin* OR lactacystin* OR lactivicin* OR laidlomycin* OR lanopepden* OR lasalocid* OR lavanducyanin* OR lenoremycin* OR linezolid* OR linopristin* OR lonomycin* OR lotilibcin* OR lydicamycin* OR lysocellin* OR macrolide* OR malyngolide* OR manumycin* OR methylenomycin* OR mikamycin* OR monensin* OR monensin* OR mureidomycin* OR mycolog* OR myxothiazol* OR narasin* OR negamycin* OR nybomycin* OR olaquindox* OR paldimycin* OR patulin* OR pentalenolactone* OR platensimycin* OR pluramycin* OR polyactin* OR polyfungin* OR posizolid* OR pristinamycin* OR prothracarcin* OR pseudomonic-acid* OR pyrroxamycin* OR quinomycin* OR quinupristin* OR radezolid* OR radicicol* OR ranbezolid* OR simaomicin* OR simocyclinone* OR spectinomycin* OR squalamine* OR streptogramin* OR streptovitacin* OR tedizolid* OR terdecamycin* OR tetracycline* OR tetronasin* OR tetronomycin* OR tetroxoprim* OR thiolactomycin* OR tibezonium-iodide* OR tizoxanide* OR toyocamycin* OR toyocamycin* OR trichostatic-acid* OR trichostatin* OR trimethoprim* OR trimethoprim* OR triostin* OR trospectomycin* OR tuftsin* OR tuftsin* OR tutofusin* OR urdamycin* OR validamycin* OR vernamycin* OR virginiae-butanolide* OR virginiamycin* OR volpristin* OR zibrofusidic-acid* OR zorbamycin* OR fluoroquinolon* OR quinolon* OR multidrug* OR multi-drug* OR methicillin* OR ticarcillin* OR ampicillin* OR ciprofloxacin* OR cefprozil* OR cefaclor* OR amoxicillin* OR streptomycin*) ADJ10 (sensitiv* OR susceptib*)) OR resistan*).ab,ti.) AND (exp "Streptococcus pneumoniae"/ OR exp "Pneumococcal Infections"/ OR exp "Haemophilus influenzae"/ OR exp "Haemophilus Infections"/ OR exp "Meningitis, Bacterial"/ OR exp "Pneumonia, Bacterial"/ OR "otitis media"/ OR ((Streptococc* ADJ3 pneumon*) OR (Haemoph* ADJ3 (influen* OR meningit*)) OR ((respirator* OR food-born* OR foodborn*) ADJ3 pathogen*) OR ((enteric* OR typhoid*) ADJ3 fever*) OR pneumococc* OR (bacteri* ADJ3 meningit*) OR (otitis ADJ3 (media OR infect*)) OR ((Communit* OR bacter*) ADJ3 pneumonia*)).ab,ti.) AND ("Climate"/ OR Seasons/ OR Weather/ OR ((time ADJ3 series*) OR season* OR Autumn* OR spring OR summer* OR winter* OR meteorolog* OR weather* OR climate* OR ((temporal* OR month* ) ADJ6 (variat* OR higher* OR lower* OR compar* OR divers* OR risk OR fluctuat* OR peak OR pattern* OR dynamic* OR trend* OR monitor* OR decline* OR decrease* OR increase* OR incline* OR change* OR associat*)) OR (throughout ADJ3 year) ).ab,ti.)

**Cochrane 60**

((((antibiotic* OR antibacter* OR anti-biotic* OR antiinfect* OR anti-infect* OR anti-bacter* OR antimicrob* OR anti-microb* OR penicillin* OR aminoglycosid* OR lactam* OR cephalosporin* OR fenicole* OR fluorochinono* OR macrolide* OR trimethoprim* OR tetracyclin* OR sulphamide* OR abyssomicin* OR acetomycin* OR actinorhodine* OR aditoprim* OR agglomerin* OR alafosfalin* OR aldecalmycin* OR alisamycin* OR allicin* OR ambruticin* OR ansamitocin* OR ansamycin* OR aplasmomycin* OR aristeromycin* OR asukamycin* OR atpenin* OR auricularum* OR aurograb* OR avilamycin* OR bafilomycin* OR baliz* OR baquiloprim* OR beroline* OR betafectin* OR betamipron* OR boromycin* OR borrelidin* OR brilacidin* OR butalactin* OR cadazolid* OR calcimycin* OR carbadox* OR carbazomycin* OR chloramphenicol* OR ciadox* OR cinoquidox* OR citrinin* OR concanamycin* OR coumamycin* OR cryptosporin* OR cycloheximide* OR dalfopristin* OR dealanylalahopcin* OR dioxidine* OR echinomycin* OR edeine* OR efepristin* OR emimycin* OR endusamycin* OR eperezolid* OR epetraborole* OR epiderstatin* OR epiroprim* OR ethylhydrocupreine* OR evernimicin* OR everninomicin* OR flopristin* OR fosmidomycin* OR furaquinocin* OR furazidin* OR furazolium* OR fusafungine* OR fusidate-sodium* OR fusidic-acid* OR gepotidacin* OR grisein* OR hatomamicin* OR hedamycin* OR heliomycin* OR hidamicin* OR hymeglusin* OR iclaprim* OR ikarugamycin* OR inostamycin* OR kalafungin* OR kelfiprim* OR kidamycin* OR kinamycin* OR kinamycin* OR lactacystin* OR lactivicin* OR laidlomycin* OR lanopepden* OR lasalocid* OR lavanducyanin* OR lenoremycin* OR linezolid* OR linopristin* OR lonomycin* OR lotilibcin* OR lydicamycin* OR lysocellin* OR macrolide* OR malyngolide* OR manumycin* OR methylenomycin* OR mikamycin* OR monensin* OR monensin* OR mureidomycin* OR mycolog* OR myxothiazol* OR narasin* OR negamycin* OR nybomycin* OR olaquindox* OR paldimycin* OR patulin* OR pentalenolactone* OR platensimycin* OR pluramycin* OR polyactin* OR polyfungin* OR posizolid* OR pristinamycin* OR prothracarcin* OR pseudomonic-acid* OR pyrroxamycin* OR quinomycin* OR quinupristin* OR radezolid* OR radicicol* OR ranbezolid* OR simaomicin* OR simocyclinone* OR spectinomycin* OR squalamine* OR streptogramin* OR streptovitacin* OR tedizolid* OR terdecamycin* OR tetracycline* OR tetronasin* OR tetronomycin* OR tetroxoprim* OR thiolactomycin* OR tibezonium-iodide* OR tizoxanide* OR toyocamycin* OR toyocamycin* OR trichostatic-acid* OR trichostatin* OR trimethoprim* OR trimethoprim* OR triostin* OR trospectomycin* OR tuftsin* OR tuftsin* OR tutofusin* OR urdamycin* OR validamycin* OR vernamycin* OR virginiae-butanolide* OR virginiamycin* OR volpristin* OR zibrofusidic-acid* OR zorbamycin* OR fluoroquinolon* OR quinolon* OR multidrug* OR multi-drug* OR methicillin* OR ticarcillin* OR ampicillin* OR ciprofloxacin* OR cefprozil* OR cefaclor* OR amoxicillin* OR streptomycin*) NEAR/10 (sensitiv* OR susceptib*)) OR resistan*):ab,ti) AND (((Streptococc* NEAR/3 pneumon*) OR (Haemoph* NEAR/3 (influen* OR meningit*)) OR ((respirator* OR food-born* OR foodborn*) NEAR/3 pathogen*) OR ((enteric* OR typhoid*) NEAR/3 fever*) OR pneumococc* OR (bacteri* NEAR/3 meningit*) OR (otitis NEAR/3 (media OR infect*)) OR ((Communit* OR bacter*) NEAR/3 pneumonia*)):ab,ti) AND (((time NEAR/3 series*) OR season* OR Autumn* OR spring OR summer* OR winter* OR meteorolog* OR weather* OR climate* OR ((temporal* OR month* ) NEAR/6 (variat* OR higher* OR lower* OR compar* OR divers* OR risk OR fluctuat* OR peak OR pattern* OR dynamic* OR trend* OR monitor* OR decline* OR decrease* OR increase* OR incline* OR change* OR associat*)) OR (throughout NEAR/3 year) OR periodicity):ab,ti)

**Web of science 783**

TS=(((((antibiotic* OR antibacter* OR anti-biotic* OR antiinfect* OR anti-infect* OR anti-bacter* OR antimicrob* OR anti-microb* OR penicillin* OR aminoglycosid* OR lactam* OR cephalosporin* OR fenicole* OR fluorochinono* OR macrolide* OR trimethoprim* OR tetracyclin* OR sulphamide* OR abyssomicin* OR acetomycin* OR actinorhodine* OR aditoprim* OR agglomerin* OR alafosfalin* OR aldecalmycin* OR alisamycin* OR allicin* OR ambruticin* OR ansamitocin* OR ansamycin* OR aplasmomycin* OR aristeromycin* OR asukamycin* OR atpenin* OR auricularum* OR aurograb* OR avilamycin* OR bafilomycin* OR baliz* OR baquiloprim* OR beroline* OR betafectin* OR betamipron* OR boromycin* OR borrelidin* OR brilacidin* OR butalactin* OR cadazolid* OR calcimycin* OR carbadox* OR carbazomycin* OR chloramphenicol* OR ciadox* OR cinoquidox* OR citrinin* OR concanamycin* OR coumamycin* OR cryptosporin* OR cycloheximide* OR dalfopristin* OR dealanylalahopcin* OR dioxidine* OR echinomycin* OR edeine* OR efepristin* OR emimycin* OR endusamycin* OR eperezolid* OR epetraborole* OR epiderstatin* OR epiroprim* OR ethylhydrocupreine* OR evernimicin* OR everninomicin* OR flopristin* OR fosmidomycin* OR furaquinocin* OR furazidin* OR furazolium* OR fusafungine* OR fusidate-sodium* OR fusidic-acid* OR gepotidacin* OR grisein* OR hatomamicin* OR hedamycin* OR heliomycin* OR hidamicin* OR hymeglusin* OR iclaprim* OR ikarugamycin* OR inostamycin* OR kalafungin* OR kelfiprim* OR kidamycin* OR kinamycin* OR kinamycin* OR lactacystin* OR lactivicin* OR laidlomycin* OR lanopepden* OR lasalocid* OR lavanducyanin* OR lenoremycin* OR linezolid* OR linopristin* OR lonomycin* OR lotilibcin* OR lydicamycin* OR lysocellin* OR macrolide* OR malyngolide* OR manumycin* OR methylenomycin* OR mikamycin* OR monensin* OR monensin* OR mureidomycin* OR mycolog* OR myxothiazol* OR narasin* OR negamycin* OR nybomycin* OR olaquindox* OR paldimycin* OR patulin* OR pentalenolactone* OR platensimycin* OR pluramycin* OR polyactin* OR polyfungin* OR posizolid* OR pristinamycin* OR prothracarcin* OR pseudomonic-acid* OR pyrroxamycin* OR quinomycin* OR quinupristin* OR radezolid* OR radicicol* OR ranbezolid* OR simaomicin* OR simocyclinone* OR spectinomycin* OR squalamine* OR streptogramin* OR streptovitacin* OR tedizolid* OR terdecamycin* OR tetracycline* OR tetronasin* OR tetronomycin* OR tetroxoprim* OR thiolactomycin* OR tibezonium-iodide* OR tizoxanide* OR toyocamycin* OR toyocamycin* OR trichostatic-acid* OR trichostatin* OR trimethoprim* OR trimethoprim* OR triostin* OR trospectomycin* OR tuftsin* OR tuftsin* OR tutofusin* OR urdamycin* OR validamycin* OR vernamycin* OR virginiae-butanolide* OR virginiamycin* OR volpristin* OR zibrofusidic-acid* OR zorbamycin* OR fluoroquinolon* OR quinolon* OR multidrug* OR multi-drug* OR methicillin* OR ticarcillin* OR ampicillin* OR ciprofloxacin* OR cefprozil* OR cefaclor* OR amoxicillin* OR streptomycin*) NEAR/9 (sensitiv* OR susceptib*)) OR resistan*)) AND (((Streptococc* NEAR/2 pneumon*) OR (Haemoph* NEAR/2 (influen* OR meningit*)) OR ((respirator* OR food-born* OR foodborn*) NEAR/2 pathogen*) OR ((enteric* OR typhoid*) NEAR/2 fever*) OR pneumococc* OR (bacteri* NEAR/2 meningit*) OR (otitis NEAR/2 (media OR infect*)) OR ((Communit* OR bacter*) NEAR/2 pneumonia*))) AND (((time NEAR/2 series*) OR season* OR Autumn* OR spring OR summer* OR winter* OR meteorolog* OR weather* OR climate* OR ((temporal* OR month* ) NEAR/5 (variat* OR higher* OR lower* OR compar* OR divers* OR risk OR fluctuat* OR peak OR pattern* OR dynamic* OR trend* OR monitor* OR decline* OR decrease* OR increase* OR incline* OR change* OR associat*)) OR (throughout NEAR/2 year) OR "periodicity")))

**Biosis Ovid** – not used, since this database is not available for us (anymore)

((((antibiotic* OR antibacter* OR anti-biotic* OR antiinfect* OR anti-infect* OR anti-bacter* OR antimicrob* OR anti-microb* OR penicillin* OR aminoglycosid* OR lactam* OR cephalosporin* OR fenicole* OR fluorochinono* OR macrolide* OR trimethoprim* OR tetracyclin* OR sulphamide* OR abyssomicin* OR acetomycin* OR actinorhodine* OR aditoprim* OR agglomerin* OR alafosfalin* OR aldecalmycin* OR alisamycin* OR allicin* OR ambruticin* OR ansamitocin* OR ansamycin* OR aplasmomycin* OR aristeromycin* OR asukamycin* OR atpenin* OR auricularum* OR aurograb* OR avilamycin* OR bafilomycin* OR baliz* OR baquiloprim* OR beroline* OR betafectin* OR betamipron* OR boromycin* OR borrelidin* OR brilacidin* OR butalactin* OR cadazolid* OR calcimycin* OR carbadox* OR carbazomycin* OR chloramphenicol* OR ciadox* OR cinoquidox* OR citrinin* OR concanamycin* OR coumamycin* OR cryptosporin* OR cycloheximide* OR dalfopristin* OR dealanylalahopcin* OR dioxidine* OR echinomycin* OR edeine* OR efepristin* OR emimycin* OR endusamycin* OR eperezolid* OR epetraborole* OR epiderstatin* OR epiroprim* OR ethylhydrocupreine* OR evernimicin* OR everninomicin* OR flopristin* OR fosmidomycin* OR furaquinocin* OR furazidin* OR furazolium* OR fusafungine* OR fusidate-sodium* OR fusidic-acid* OR gepotidacin* OR grisein* OR hatomamicin* OR hedamycin* OR heliomycin* OR hidamicin* OR hymeglusin* OR iclaprim* OR ikarugamycin* OR inostamycin* OR kalafungin* OR kelfiprim* OR kidamycin* OR kinamycin* OR kinamycin* OR lactacystin* OR lactivicin* OR laidlomycin* OR lanopepden* OR lasalocid* OR lavanducyanin* OR lenoremycin* OR linezolid* OR linopristin* OR lonomycin* OR lotilibcin* OR lydicamycin* OR lysocellin* OR macrolide* OR malyngolide* OR manumycin* OR methylenomycin* OR mikamycin* OR monensin* OR monensin* OR mureidomycin* OR mycolog* OR myxothiazol* OR narasin* OR negamycin* OR nybomycin* OR olaquindox* OR paldimycin* OR patulin* OR pentalenolactone* OR platensimycin* OR pluramycin* OR polyactin* OR polyfungin* OR posizolid* OR pristinamycin* OR prothracarcin* OR pseudomonic-acid* OR pyrroxamycin* OR quinomycin* OR quinupristin* OR radezolid* OR radicicol* OR ranbezolid* OR simaomicin* OR simocyclinone* OR spectinomycin* OR squalamine* OR streptogramin* OR streptovitacin* OR tedizolid* OR terdecamycin* OR tetracycline* OR tetronasin* OR tetronomycin* OR tetroxoprim* OR thiolactomycin* OR tibezonium-iodide* OR tizoxanide* OR toyocamycin* OR toyocamycin* OR trichostatic-acid* OR trichostatin* OR trimethoprim* OR trimethoprim* OR triostin* OR trospectomycin* OR tuftsin* OR tuftsin* OR tutofusin* OR urdamycin* OR validamycin* OR vernamycin* OR virginiae-butanolide* OR virginiamycin* OR volpristin* OR zibrofusidic-acid* OR zorbamycin* OR fluoroquinolon* OR quinolon* OR multidrug* OR multi-drug* OR methicillin* OR ticarcillin* OR ampicillin* OR ciprofloxacin* OR cefprozil* OR cefaclor* OR amoxicillin* OR streptomycin*) ADJ10 (sensitiv* OR susceptib*)) OR resistan*).ab,ti.) AND ((Campylobacter* OR Salmonell* OR "Escherichia coli" OR e-coli OR (Streptococc* ADJ3 pneumon*) OR (Haemoph* ADJ3 (influen* OR meningit*)) OR ((respirator* OR food-born* OR foodborn*) ADJ3 pathogen*) OR ((enteric* OR typhoid*) ADJ3 fever*) OR pneumococc* OR (bacteri* ADJ3 meningit*) OR (otitis ADJ3 (media OR infect*)) OR ((Communit* OR bacter*) ADJ3 pneumonia*)).ab,ti.) AND (" Climatology"/ OR ((time ADJ3 series*) OR season* OR Autumn* OR spring OR summer* OR winter* OR meteorolog* OR weather* OR climate* OR ((temporal* OR month* ) ADJ6 (variat* OR higher* OR lower* OR compar* OR divers* OR risk OR fluctuat* OR peak OR pattern* OR dynamic* OR trend* OR monitor* OR decline* OR decrease* OR increase* OR incline* OR change* OR associat*)) OR (throughout ADJ3 year) ).ab,ti.)

**Google scholar**

"antibiotic|antibacterial|biotic sensitivity|susceptibility|resistance" Streptococca|Haemophilae|"respiratory|foodborne pathogens" "time series"|season|seasonal|weather|climate|"temporal variation|fluctuation"
