# Supplementary material for: Seasonality of antimicrobial resistance rates in respiratory bacteria: A systematic review and meta-analysis
Source: PLoS One. 2019 Aug 15;14(8):e0221133. doi: 10.1371/journal.pone.0221133 (PMC6695168; doi:10.1371/journal.pone.0221133)
Supplement: S2 Text — (DOCX) [file pone.0221133.s003.docx]

# S2 Text. Modified version of the Newcastle-Ottawa Scale (NOS) for cross-sectional studies

Quality assessment was based on a adapted version from the Newcastle-Ottawa Quality Assessment Scale for cross-sectional studies by [1]. The three factors considered were:

- **Sample selection criteria (maximum 4 points):**

1. *Representativeness of the sample/sample size*
2. Truly representative of the average in the target population (random sampling) and justified sample size based on population characteristics (i.e. farms production, hospitals, slaughterhouses, river length). **
3. Somewhat representative of the average in the target population (convenience sampling or other sampling method), justified sample size based on population characteristics. *****
4. No description of the sampling strategy/sample size not justified.
5. *Study time period*
   1. The time period was satisfactory (i.e. monthly observations) making possible the categorization of the four seasons (summer, winter, spring and autumn) **
   2. The time-period was partly satisfactory making possible the categorization of two seasons (including cold and warm seasons). *
   3. The time-period was unsatisfactory making difficult the categorization of seasons.

- **Comparability on the basis of the design or analysis (maximum 2 points):**

1. *Comparability of the population (control of confounders):*
   1. Population is comparable throughout the time-period of study (have the same characteristics) and adjusted for at least an important factor (i.e. seasonal prevalence of resistant strains, population, year). **
   2. Population is not comparable throughout the time-period of study (different characteristics) but adjusted for at least an important factor. *
   3. Population is not comparable and not adjusted.

- **Outcome (maximum 2 points):**

1. *Assessment of the exposure:*
   1. Medical records or laboratory assessment with a validated antimicrobial susceptibility testing and interpretation method based on standard minimal concentration break points*
   2. Self-report (i.e. survey, questionnaire).
   3. No description of antimicrobial susceptibility testing and interpretation method.
2. *Data analysis and statistical test:*
   1. Total number of samples, frequencies and proportion of susceptible and non-susceptible strains are clearly described, as well as the statistical test used to analyze the data including p-values. *****
   2. The data described is incomplete, the statistical test is not appropriate or not well describe.

**References**

1. Modesti PA, Reboldi G, Cappuccio FP, Agyemang C, Remuzzi G, Rapi S, et al. Panethnic Differences in Blood Pressure in Europe: A Systematic Review and Meta-Analysis. PLOS ONE. 2016;11(1):e0147601. doi: 10.1371/journal.pone.0147601.
