# Supplementary material for: Seasonality of antimicrobial resistance rates in respiratory bacteria: A systematic review and meta-analysis
Source: PLoS One. 2019 Aug 15;14(8):e0221133. doi: 10.1371/journal.pone.0221133 (PMC6695168; doi:10.1371/journal.pone.0221133)
Supplement: S2 Table — (DOCX) [file pone.0221133.s004.docx]

# S2 Table. Quality assessment of included studies (n =13)

A cut-off of NOS score of 7 or more to considered as “good quality” was used. This criterion was based on Quality improvement intervention to Adress Health Imparities report by McPheeters et al. 2012; appendix G, page 103-104. Tool used and criteria for quality assessment is provided in supplement 2. Bold letter indicate author that were contacted for additional search studies.

| **First autor** | **Study year** | **Sample Criteria** | | **Comparability** | **Outcome** | | **Total score** | **Quality assessment interpretation** |
| --- | --- | --- | --- | --- | --- | --- | --- | --- |
|  |  | **Representativeness of the sample** | **Study time period** | **Control of confounders** | **Assessment of the exposure** | **Data analysis and statistical testing** |  |  |
| ***Streptococcus pneumoniae*** | |  |  |  |  |  |  |  |
| Stacevičiene et al. | 2016 | * | ** | ** | * | * | 7 | Good quality |
| Baquero et al. | 1999 | * | ** | - | * | * | 5 | Fair quality |
| Boken et al. | 1995 | * | * | * | * | * | 5 | Fair quality |
| Vardhan et al. | 2003 | ** | ** | - | * | - | 5 | Fair quality |
| Siripongpreeda et al. | 2010 | * | ** | * | * | * | 6 | Fair quality |
| Albanese et al. | 2001 | * | * | ** | * | * | 6 | Fair quality |
| Hoberman et al. | 2005 | * | ** | ** | * | * | 7 | Good quality |
| Guevara et al. | 2008 | ** | * | * | * | * | 6 | Fair quality |
| Marco et al. | 2000 | * | * | * | * | * | 5 | Fair quality |
| Dagan et al. | 2008 | ** | ** | ** | * | * | 8 | Good quality |
| Tam et al. | 2015 | * | ** | * | - | * | 5 | Fair quality |
| Mϋhlemann et al. | 2006 | * | * | ** | * | * | 6 | Fair quality |
| ***Haemophilus influenzae*** | |  |  |  |  |  |  |  |
| Marchisio et al. | 2001 | ** | * | * | * | * | 6 | Fair quality |
| Hashida et al. | 2008 | * | * | * | * | * | 5 | Fair quality |
