# Supplementary material for: Seasonality of antimicrobial resistance rates in respiratory bacteria: A systematic review and meta-analysis
Source: PLoS One. 2019 Aug 15;14(8):e0221133. doi: 10.1371/journal.pone.0221133 (PMC6695168; doi:10.1371/journal.pone.0221133)
Supplement: S3 Table — (DOCX) [file pone.0221133.s005.docx]

# S3 Table. Description of studies describing seasonality of antimicrobial resistance rates in respiratory bacteria

| **Study region** | **Citation** | **Study description** | **Seasons** | **Season definition** | **AMR Pattern** | **Reported resistance levels** | | **Resistance ratio ^ŧ^** | **Relevant findings** | **Seasonal AMR variation explanation** |
| --- | --- | --- | --- | --- | --- | --- | --- | --- | --- | --- |
|  |  |  |  |  |  | **n/N** | **%** |  |  |  |
| America | Guevara et al., (2008) | 952 children with otitis media aged 3-144 months participated to determine the seasonal respiratory pathogen and AMR distribution. | Wet | May - Nov | PEN | 6/122 | 5.0 | Ref. | *S. pneumoniae* and *H. influenzae* were the most common respiratory pathogens. There was non-statistical significance trend of higher frequency of these two pathogens between seasons. Penicillin non-susceptible *S. pneumoniae* was more frequent in the rainy seasons (38%) than in the dry seasons (18%) (P=0.003; OR: 2.94; 95% Confidence interval: 1.4-6.45). | Factors associated with an increase number on antibiotic consumption such as influenzae season occurring in the rainy season. Besides, increase of admission in hospital with *S. pneumoniae* during the rainy seasons, suggesting more use of antibiotics in that time of the year. |
|  |  |  |  |  | CEP | 0/43 | 0.0 |  |  |  |
|  |  |  |  |  | MC | 9/78 | 8.0 |  |  |  |
|  |  |  |  |  | TM/SUL | 41/144 | 36.0 |  |  |  |
|  |  |  | Dry | Dec - Apr | PEN | 7/74 | 9.5 | 0.46 |  |  |
|  |  |  |  |  | CEP | 0/100 | 0.0 | - |  |  |
|  |  |  |  |  | MC | 7/65 | 9.0 | 0.22 |  |  |
|  |  |  |  |  | TM/SUL | 51/98 | 52.0 | 0.63 |  |  |
|  | Boken et al., (1995) | Nasopharyngel cultures of *S. pneumoniae* from 59 children aged 2 to 24 months attending in a clinical care to determine prevalence and risk factors for infections with penecillin-resistant pneumococcal isolates. | Spring | Apr | PEN | 17/59 | 28.8 | Ref. | Pneumococcal isolates mainly belonged to serotypes 23.19.6 and 11. Associated risk factor to carriage of penicillin resistant isolates was increasing in age (until 14 months), and exposure to antibiotics 2 months previous culture (RR: 2.1; 95% CI: 0.72-6.06 in April. and RR: 1; 95CI: 0.38-2.6 in August). No association was seen with gender. | Cofactor of temporal variation is the increased AMU during winter season (prolonged and intense). Besides, seasonal viral infections are more likely to lead to otitis media and subsequent antibiotic use in the winter season. Since fewer respiratory infections happens in summer, both AMU and AMR should diminish. |
|  |  |  | Autumn | Aug |  | 2/71 | 2.8 | 0.10 |  |  |
|  | Albanese et al., (2002) | 2094 patients of any age with invasive *S. pneumonia* disease identified in a city area to describe the epidemiology of these infections. | respiratory | Nov - Apr | PEN | - | 8.0 | Ref. | Higher proportion of penicillin-resistance strains occurred in the respiratory season (winter/spring) (P=0.03). In particular, patients aged 0-4 and >65 had greater proportion of resistance strains during the respiratory season. An adjusted OR of 1.6 (1.0-2.4) was determined for the respiratory season comparing with OR of 1 for non-respiratory season. | Seasonal difference was correlated with trend in AMU, where higher use occurred in the winter. Besides, differential antibiotic use may play a role in differences between age and geographical location. |
|  |  |  | Non-respiratory | May - Oct |  | - | 5.0 | 0.60 |  |  |
|  | Tam et al., (2015) | 253 cases of invasive pneumococcal disease in children aged ≤ 5 years to determine its association with penicillin susceptible rates with higher non-susceptible rates in the winter season. | Winter | Jan - Mar | PEN | 40 | 16.0 | Ref. | There was a significant variation in pneumococcal cases by months and seasons, with autumn and winter with higher incidence. There was no significant association between seasonality and age (P=0.3). Penicillin non-susceptible was highest in winter (15.8), and lowest in the summer (9.1%). but differences were not significant. | Seasonal difference due to increased AMU during cold months on pneumococcal carriage prevalence and density. Besides, seasonal prescribing practices at community level would play an important role. |
|  |  |  | Spring | Apr - Jun |  | 28 | 11.0 | 0.69 |  |  |
|  |  |  | Summer | Jul - Sep |  | 23 | 9.0 | 0.56 |  |  |
|  |  |  | Autumn | Oct - Dec |  | 30 | 12.0 | 0.75 |  |  |
|  | Hoberman et al., (2005) | 629 children with acute otitis media cases were estudied to determine whether seasonal variation exists among *S. pneumoniae* isolates. | Winter | Dec - Feb | PEN | 11/51 | 21.6 | Ref. | Resistant infections increased over time from 0% to 58% from 1996 to 2003. Resistant acute otitis infections occurred at higher rate as winter progressed (P=0.03). Resistant proportion was much higher in spring than winter (P=0.03). | Seasonal variation related to day-care attendance. Children may increase the chance to acquire viral respiratory infection when exposed to other children, and thus develop acute otitis, especially in winters. Subsequently. AMU increase and therefore selective pressure in such settings. |
|  |  |  | Spring | Mar - May |  | 23/73 | 31.5 | 1.46 |  |  |
|  |  |  | Summer | Jun - Aug |  | 1/7 | 10.0 | 0.46 |  |  |
|  |  |  | Autumn | Sep - Nov |  | 3/23 | 9.7 | 0.45 |  |  |
| Europe | Baquero et al., (1996) | Data of 1113 *S. pneumoniae* isolates was studied to describe its susceptibility profile in a nationwide antimicrobial surveillance program. | Winter | Dec - Feb | PEN | 154/357 | 43.1 | Ref. | The prevalence of resistance was about 10% for CEP, 25% for animopenicillins, >30% for PEN and MC, and 46% for cefuroxime. Statistically significant (P<0.05) difference between seasons was found, with higher rates of resistant to β-lactams in summer than in winter. No AMR seasonality was observed for macrolides. | AMU would be reason for the seasonal variation in pencillin-resistance isolates in this study. Besides, it is suggested that the spread of resistant clones might also play a role. |
|  |  |  | Spring | Mar - May |  | 96/321 | 29.9 | 0.70 |  |  |
|  |  |  | Summer | Jun - Aug |  | 62/150 | 41.3 | 0.95 |  |  |
|  |  |  | Autumn | Sep - Nov |  | 89/286 | 31.1 | 0.72 |  |  |
|  | Dagan et al., (2008) | A total of 236.466 prescriptions and 3609 *S. pneumoniae* cases in children were used to determine seasonal variation in antibiotic prescription and to compare this with seasonal resistance variation. Data from care clinics located in two regions (urban and rural). | Cold | Oct - Mar | PEN | 382/888 | 43.0 | Ref. | Antibiotic prescription rates decreased during warm months from 36% to 15% between the two regions (P=0.001). In rural cases, higher resistance rates were observed significant when adjusted for age, ethnic group, study year, history of antibiotic use and serotype. In urban cases was determined an association between each monthly increase in 10 prescription/1000 children with increase of odds to 1.05 (95%CI: 1.03-1.07), 1.04 (95%CI: 1.02-1.05) and 1.04 for penicillins (95%CI: 1.02-1.06), erythromicin and MDR, respectively. These associations were not seen in rural cases. Yearly reduction in antibiotic prescriptions during the warm months was significantly associated with reduction in AMR among pneumococcal isolates. | Difference seasonal variation between urban and rural isolates would be a response on differences in AMU. |
|  |  |  |  |  | MC | 258/888 | 29.0 |  |  |  |
|  |  |  |  |  | MDR | 213/888 | 24.0 |  |  |  |
|  |  |  | Warm | Apr - Sep | PEN | 148/512 | 29.0 | 0.67 |  |  |
|  |  |  |  |  | MC | 102/512 | 20.0 | 0.69 |  |  |
|  |  |  |  |  | MDR | 77/512 | 15.0 | 0.63 |  |  |
|  |  |  | ^ꝉ^ Cold | Oct - Mar | PEN | 364/1399 | 26.0 | Ref. |  |  |
|  |  |  |  |  | MC | 224/1399 | 16.0 |  |  |  |
|  |  |  |  |  | MDR | 280/1399 | 20.0 |  |  |  |
|  |  |  | ^ꝉ^ Warm | Apr - Sep | PEN | 169/806 | 21.0 | 0.81 |  |  |
|  |  |  |  |  | MC | 137/806 | 17.0 | 1.06 |  |  |
|  |  |  |  |  | MDR | 153/806 | 19.0 | 0.95 |  |  |
|  | Marco et al., (2000) | 1113 isolates of *S. pneumoniae* from patients (children. adults) were analyzed to determine de impact of geographical site. Serotype, season, age on AMR pattern. | Winter | Dec - Feb | PEN | 115/357 | 32.3 | Ref. | Resistance isolates depends on geographical region, serotype, patient age and sample origin. Resistance rates of pneumococcal to penicillin and cefuroxime were significantly higher in children than in adults. The prevalence of resistance to β-lactams (oral antibiotics) showed seasonal pattern, with higher rates in summer than in winter. | Seasonality for oral β-lactams, and parenteral third generation CEP would be the result of seasonal variability of isolation of serotypes. |
|  |  |  | Spring | Mar - May |  | 28/113 | 24.8 | 0.75 |  |  |
|  |  |  | Summer | Jun - Aug |  | 47/150 | 31.1 | 0.96 |  |  |
|  |  |  | Autumn | Sep - Nov |  | 65/286 | 22.7 | 0.69 |  |  |
|  |  |  | Winter | Dec - Feb | CEP | 91/357 | 25.4 | Ref. |  |  |
|  |  |  | Spring | Mar - May |  | 23/113 | 20.6 | 0.76 |  |  |
|  |  |  | Summer | Jun - Aug |  | 37/150 | 24.4 | 0.90 |  |  |
|  |  |  | Autumn | Sep - Nov |  | 56/286 | 19.6 | 0.75 |  |  |
|  | Stacevičiene et al., (2016) | 900 participants from two hospitals were studied to evaluate the circulation of *S. pneumoniae* serotypes and AMR pattern among children with respiratory tract infection. | Winter | Dec - Feb | MDR | 32/67 | 47.8 | Ref. | Pneumococcal isolates were resistant to penicillin. Erythromycin, clindamycin and TM/SUL. Geographical variation of resistance isolates within cities. Age, sex, attendance of day care and previous AMU were not significantly associated with resistant pneumococcal isolates. When having summer as reference, MDR isolates had an OR of 1.71 (95%CI 1.02-1.36. P=0.02) in Spring, an OR of 1.09 (95%CI 0.95-1.27. P=0.22) in Autumn, and an OR of 1.15 (95%CI 0.99-1.34. P=0.06). | Serotype distribution might play a role in the seasonality of resistant isolates. Serotypes 6, 9, 14 and 23 accounted for most AMR in pneumococcal isolates. |
|  |  |  | Spring | Mar - May |  | 64/136 | 47.1 | 0.99 |  |  |
|  |  |  | Summer | Jun - Aug |  | 9/32 | 28.1 | 0.86 |  |  |
|  |  |  | Autumn | Sep - Nov |  | 54/132 | 40.9 | 0.59 |  |  |
|  | Vardhan & Allen (2003) | 549 patients with penicillin-resistant pneumococci were studied to examine its relation with sex, age, seasons, sample origin, serotype and AMR pattern. | Winter | Dec - Feb | PEN | 195 | 35.5 | Ref. | Changing of serotypes was associated with changes in AMR patterns. Seasonal incidence of penicillin-resistant isolates occurred in the winter months. Most of patients (46%) received penicillin treatment before first isolation of resistant isolates. | Seasonal variation might result from previous use of antibiotics at community - level. Besides, the changing serotypes have influence on AMR patterns. |
|  |  |  | Spring | Mar - May |  | 143 | 26.0 | 0.7 |  |  |
|  |  |  | Summer | Jun - Aug |  | 121 | 22.0 | 0.6 |  |  |
|  |  |  | Autumn | Sep - Nov |  | 90 | 16.4 | 0.5 |  |  |
|  | Marchisio et al., (2001) | 1580 healthy children aged 1 - 7 years sampled during two seasons were studied to investigate the influence of season on the prevalence of respiratory pathogens. | Spring | Oct - Nov | PEN | 3/75 | 4.0 | Ref. | There was a variation in isolation of *S. pneumoniae* and *H. influenzae* between seasons. Colonization with two or more pathogens increased from 9.1% in spring to 17.3% in autumn (P=0.04). Seasonal variation occurred in the prevalence of respiratory pathogens in healthy children. Although, the difference was slight and had limited clinical relevance. | Study limited by few sampling points and biotyping, which would explain better the seasonal variation of respiratory bacteria. However, previous respiratory infections in spring was associated with *H. influenza* due to viral infections during winter, which might enhance bacterial adherence to the nasopharynx. |
|  |  |  | Autumn | Apr - May |  | 1/60 | 1.7 | 0.71 |  |  |
|  |  |  | Spring | Oct - Nov | MC | 28/75 | 37.3 | Ref. |  |  |
|  |  |  | Autumn | Apr - May |  | 24/60 | 40.0 | 1.07 |  |  |
|  |  |  | Spring^*^ | Oct - Nov | PEN | 29/288 | 10.1 | Ref. |  |  |
|  |  |  | Autumn^*^ | Apr - May |  | 12/206 | 5.8 | 0.58 |  |  |
| East Asia | Siripongpreeda et al., (2010) | 109 pneumococcal cases in children aged 2 to 5 years were studied to assess potential clinical difference between penicillin resistant with susceptible isolates. | Winter | Nov - Feb | PEN | 11/27 | 40.7 | Ref. | Most of patients with PRSP and PSSP were treat with 3rd generation CEP, penicillin, vancomycin and carbapenem. The higher number of IPD was observed in January and July in both group (resistant and non-resistant to penicillin). | Seasonal variation would be due to higher number of pneumococcal cases occurring in January and July, which are the cooler months and rainy months, respectively. Subsequently, higher AMU. Most common empiric antibiotics was third generation CEP. |
|  |  |  | Summer | Mar - May |  | 3/10 | 30 | 0.7 |  |  |
|  |  |  | Wet | Jun - Oct |  | 3/18 | 16.7 | 0.4 |  |  |
| Western Pacific | Hashida et al., (2008) | 363 samples from children attended in a day care center with respiratory infections were studied to investigate AMR-relate genetic characteristics and the turnover of nasopharyngeal *H. influenzae* carriage | Summer ^*^ | Jul | PEN | 34/184 | 18.5 | 0.9 | The rate of nasopharyngeal carriage of *H. influenzae* was significantly increased in winter (56.4%) than in summer (38.6%). However, it was not significant difference in AMR rates against ampicillin between seasons. | Seasonal variation was explained with increased risk of recurrent otitis media and thus higher changes to be treated with antibiotics. |
|  |  |  | Winter ^*^ | Feb |  | 30/179 | 16.8 | Ref. |  |  |

**^ŧ^** Resistance ratio calculated with winter as reference group.^*^ = Comparison of *H. influenzae* resistance rates among seasons. ^ꝉ^ = comparison of group of children in rural areas. Ref. indicate the reference group, thus resistance rate = 1. PEN= pencillins, CEP= cephalosporins, TM/SUL= trimethoprim/sulphamides. AMR = antimicrobial resistance. AMU= antimicrobial use.
