# Supplementary material for: Seasonality of antimicrobial resistance rates in respiratory bacteria: A systematic review and meta-analysis
Source: PLoS One. 2019 Aug 15;14(8):e0221133. doi: 10.1371/journal.pone.0221133 (PMC6695168; doi:10.1371/journal.pone.0221133)
Supplement: S4 Table — (DOCX) [file pone.0221133.s006.docx]

# S4 Table. Description of study weights in each meta-analysis

1. **Meta-analysis of all studies describing the seasonality of AMR rates in *S. pneumoniae* isolates to all antibiotics, winter as reference group.**

| **Season** | **Study** | **OR** | **95% CI** | | **% Weight** |
| --- | --- | --- | --- | --- | --- |
|  |  |  | **Lower** | **Upper** |  |
| ***All seasons*** | Staceviciene | 0.8 | 0.4 | 1.4 | 2.1 |
|  | Staceviciene | 1.0 | 0.5 | 1.7 | 2.2 |
|  | Staceviciene | 0.4 | 0.2 | 1.1 | 0.9 |
|  | Baquero | 0.6 | 0.4 | 0.8 | 6.4 |
|  | Baquero | 0.6 | 0.4 | 0.8 | 6.1 |
|  | Baquero | 0.9 | 0.6 | 1.4 | 4.6 |
|  | Vardhan | 0.6 | 0.4 | 1.0 | 3.2 |
|  | Vardhan | 0.5 | 0.3 | 0.9 | 2.7 |
|  | Vardhan | 0.4 | 0.2 | 0.7 | 1.9 |
|  | Siripongpreeda | 0.6 | 0.1 | 4.0 | 0.2 |
|  | Siripongpreeda | 0.3 | 0.1 | 1.4 | 0.3 |
|  | Hoberman | 1.7 | 0.7 | 3.8 | 1.1 |
|  | Hoberman | 0.4 | 0.0 | 3.5 | 0.2 |
|  | Hoberman | 0.4 | 0.1 | 1.5 | 0.4 |
|  | Tam | 0.7 | 0.3 | 2.0 | 0.8 |
|  | Tam | 0.6 | 0.2 | 1.8 | 0.7 |
|  | Tam | 0.5 | 0.2 | 1.5 | 0.7 |
|  | Marco | 0.6 | 0.4 | 0.8 | 5.0 |
|  | Marco | 0.6 | 0.4 | 0.9 | 4.7 |
|  | Marco | 0.6 | 0.5 | 0.9 | 6.0 |
|  | Marco | 0.7 | 0.4 | 1.1 | 3.3 |
|  | Marco | 0.7 | 0.4 | 1.2 | 2.5 |
|  | Marco | 0.7 | 0.5 | 0.9 | 6.5 |
|  | Marco | 0.7 | 0.4 | 1.1 | 2.9 |
|  | Marco | 0.7 | 0.4 | 1.1 | 2.8 |
|  | Marco | 0.7 | 0.5 | 1.1 | 3.6 |
|  | Marco | 1.0 | 0.6 | 1.8 | 2.1 |
|  | Marco | 0.4 | 0.2 | 1.1 | 0.9 |
|  | Marco | 0.7 | 0.5 | 1.1 | 3.9 |
|  | Marco | 0.8 | 0.5 | 1.2 | 3.8 |
|  | Marco | 1.1 | 0.7 | 1.6 | 3.9 |
|  | Marco | 1.0 | 0.7 | 1.5 | 4.6 |
|  | Marco | 1.1 | 0.6 | 1.8 | 2.7 |
|  | Marco | 0.6 | 0.3 | 1.2 | 1.5 |
|  | Marco | 1.0 | 0.7 | 1.5 | 4.7 |
|  | **Pooled OR** | **0.7** | **0.6** | **0.8** | **100.0** |
| ***Spring*** | Staceviciene | 1.0 | 0.5 | 1.7 | 7.0 |
|  | Baquero | 0.6 | 0.4 | 0.8 | 23.7 |
|  | Vardhan | 0.6 | 0.4 | 1.0 | 10.6 |
|  | Hoberman | 1.7 | 0.7 | 3.8 | 3.5 |
|  | Tam | 0.6 | 0.2 | 1.8 | 2.2 |
|  | Marco | 0.7 | 0.4 | 1.1 | 9.6 |
|  | Marco | 0.7 | 0.4 | 1.1 | 8.9 |
|  | Marco | 0.7 | 0.5 | 1.1 | 11.9 |
|  | Marco | 1.0 | 0.6 | 1.8 | 6.8 |
|  | Marco | 0.4 | 0.2 | 1.1 | 2.9 |
|  | Marco | 0.7 | 0.5 | 1.1 | 13.1 |
|  | **Pooled OR** | **0.7** | **0.6** | **0.8** | **100.0** |
| ***Summer*** | Staceviciene | 0.4 | 0.2 | 1.1 | 4.0 |
|  | Baquero | 0.9 | 0.6 | 1.4 | 14.3 |
|  | Vardhan | 0.4 | 0.2 | 0.7 | 7.4 |
|  | Siripongpreeda | 0.6 | 0.1 | 4.0 | 1.1 |
|  | Hoberman | 0.4 | 0.0 | 3.5 | 0.8 |
|  | Tam | 0.5 | 0.2 | 1.5 | 2.9 |
|  | Marco | 0.8 | 0.5 | 1.2 | 12.5 |
|  | Marco | 1.1 | 0.7 | 1.6 | 12.7 |
|  | Marco | 1.0 | 0.7 | 1.5 | 14.2 |
|  | Marco | 1.1 | 0.6 | 1.8 | 9.7 |
|  | Marco | 0.6 | 0.3 | 1.2 | 6.1 |
|  | Marco | 1.0 | 0.7 | 1.5 | 14.5 |
|  | **Pooled OR** | **0.8** | **0.7** | **1.0** | **100.0** |
| ***Autumn*** | Staceviciene | 0.8 | 0.4 | 1.4 | 4.9 |
|  | Baquero | 0.6 | 0.4 | 0.8 | 15.9 |
|  | Vardhan | 0.5 | 0.3 | 0.9 | 6.3 |
|  | Hoberman | 0.4 | 0.1 | 1.5 | 0.9 |
|  | Tam | 0.7 | 0.3 | 2.0 | 1.7 |
|  | Marco | 0.6 | 0.4 | 0.8 | 12.6 |
|  | Marco | 0.6 | 0.4 | 0.9 | 11.7 |
|  | Marco | 0.6 | 0.5 | 0.9 | 15.6 |
|  | Marco | 0.7 | 0.4 | 1.1 | 7.7 |
|  | Marco | 0.7 | 0.4 | 1.2 | 5.8 |
|  | Marco | 0.7 | 0.5 | 0.9 | 17.1 |
|  | **Pooled OR** | **0.6** | **0.6** | **0.7** | **100.0** |

1. **Meta-analysis of all studies describing the seasonality of AMR rates in *S. pneumoniae* isolates to penicillins, winter as reference group.**

| **Season** | **Study** | **OR** | **95% CI** | **% Weight** |  |
| --- | --- | --- | --- | --- | --- |
|  |  |  | **Lower** | **Upper** |  |
| ***All seasons*** | Baquero | 0.6 | 0.4 | 0.8 | 9.2 |
|  | Baquero | 0.6 | 0.4 | 0.8 | 8.9 |
|  | Baquero | 0.9 | 0.6 | 1.4 | 6.9 |
|  | Vardhan | 0.6 | 0.4 | 1.0 | 4.9 |
|  | Vardhan | 0.5 | 0.3 | 0.9 | 4.2 |
|  | Vardhan | 0.4 | 0.2 | 0.7 | 3.0 |
|  | Siripongpreeda | 0.6 | 0.1 | 4.0 | 0.4 |
|  | Siripongpreeda | 0.3 | 0.1 | 1.4 | 0.5 |
|  | Hoberman | 1.7 | 0.7 | 3.8 | 1.8 |
|  | Hoberman | 0.4 | 0.0 | 3.5 | 0.3 |
|  | Hoberman | 0.4 | 0.1 | 1.5 | 0.7 |
|  | Tam | 0.7 | 0.3 | 2.0 | 1.2 |
|  | Tam | 0.6 | 0.2 | 1.8 | 1.2 |
|  | Tam | 0.5 | 0.2 | 1.5 | 1.1 |
|  | Marco | 0.6 | 0.4 | 0.8 | 7.4 |
|  | Marco | 0.6 | 0.4 | 0.9 | 7.0 |
|  | Marco | 0.6 | 0.5 | 0.9 | 8.7 |
|  | Marco | 0.7 | 0.4 | 1.1 | 4.5 |
|  | Marco | 0.7 | 0.4 | 1.1 | 4.3 |
|  | Marco | 0.7 | 0.5 | 1.1 | 5.4 |
|  | Marco | 0.8 | 0.5 | 1.2 | 5.7 |
|  | Marco | 1.1 | 0.7 | 1.6 | 5.9 |
|  | Marco | 1.0 | 0.7 | 1.5 | 6.8 |
|  | **Pooled OR** | **0.68** | **0.60** | **0.76** | **100.00** |
| ***Spring*** | Baquero | 0.6 | 0.4 | 0.8 | 33.7 |
|  | Vardhan | 0.6 | 0.4 | 1.0 | 15.1 |
|  | Hoberman | 1.7 | 0.7 | 3.8 | 4.9 |
|  | Tam | 0.6 | 0.2 | 1.8 | 3.2 |
|  | Marco | 0.7 | 0.4 | 1.1 | 13.6 |
|  | Marco | 0.7 | 0.4 | 1.1 | 12.7 |
|  | Marco | 0.7 | 0.5 | 1.1 | 16.9 |
|  | **Pooled OR** | **0.7** | **0.6** | **0.8** | **100.0** |
| ***Summer*** | Baquero | 0.9 | 0.6 | 1.4 | 21.2 |
|  | Vardhan | 0.4 | 0.2 | 0.7 | 11.8 |
|  | Siripongpreeda | 0.6 | 0.1 | 4.0 | 1.8 |
|  | Hoberman | 0.4 | 0.0 | 3.5 | 1.3 |
|  | Tam | 0.5 | 0.2 | 1.5 | 4.8 |
|  | Marco | 0.8 | 0.5 | 1.2 | 18.9 |
|  | Marco | 1.1 | 0.7 | 1.6 | 19.2 |
|  | Marco | 1.0 | 0.7 | 1.5 | 21.1 |
|  | **Pooled OR** | **0.8** | **0.6** | **1.0** | **100.0** |
| ***Autumn*** | Baquero | 0.6 | 0.4 | 0.8 | 24.6 |
|  | Vardhan | 0.5 | 0.3 | 0.9 | 9.7 |
|  | Hoberman | 0.4 | 0.1 | 1.5 | 1.4 |
|  | Tam | 0.7 | 0.3 | 2.0 | 2.6 |
|  | Marco | 0.6 | 0.4 | 0.8 | 19.5 |
|  | Marco | 0.6 | 0.4 | 0.9 | 18.1 |
|  | Marco | 0.6 | 0.5 | 0.9 | 24.2 |
|  | **Pooled OR** | **0.6** | **0.5** | **0.7** | **100.0** |

1. **Meta-analysis of studies done in the Northern hemisphere describing the seasonality of AMR rates in *S. pneumoniae* isolates to all antibiotics, winter as reference group.**

| **Season** | **Study** | **OR** | **95% CI** | | **% Weight** |
| --- | --- | --- | --- | --- | --- |
|  |  |  | **Lower** | **Upper** |  |
| ***All seasons*** | Staceviciene | 0.8 | 0.4 | 1.4 | 2.2 |
|  | Staceviciene | 1.0 | 0.5 | 1.7 | 2.2 |
|  | Staceviciene | 0.4 | 0.2 | 1.1 | 1.0 |
|  | Baquero | 0.6 | 0.4 | 0.8 | 6.3 |
|  | Baquero | 0.6 | 0.4 | 0.8 | 6.1 |
|  | Baquero | 0.9 | 0.6 | 1.4 | 4.6 |
|  | Vardhan | 0.6 | 0.4 | 1.0 | 3.2 |
|  | Vardhan | 0.5 | 0.3 | 0.9 | 2.8 |
|  | Vardhan | 0.4 | 0.2 | 0.7 | 1.9 |
|  | Hoberman | 1.7 | 0.7 | 3.8 | 1.2 |
|  | Hoberman | 0.4 | 0.0 | 3.5 | 0.2 |
|  | Hoberman | 0.4 | 0.1 | 1.5 | 0.4 |
|  | Tam | 0.7 | 0.3 | 2.0 | 0.8 |
|  | Tam | 0.6 | 0.2 | 1.8 | 0.8 |
|  | Tam | 0.5 | 0.2 | 1.5 | 0.7 |
|  | Marco | 0.6 | 0.4 | 0.8 | 5.0 |
|  | Marco | 0.6 | 0.4 | 0.9 | 4.7 |
|  | Marco | 0.6 | 0.5 | 0.9 | 6.0 |
|  | Marco | 0.7 | 0.4 | 1.1 | 3.3 |
|  | Marco | 0.7 | 0.4 | 1.2 | 2.6 |
|  | Marco | 0.7 | 0.5 | 0.9 | 6.4 |
|  | Marco | 0.7 | 0.4 | 1.1 | 3.0 |
|  | Marco | 0.7 | 0.4 | 1.1 | 2.8 |
|  | Marco | 0.7 | 0.5 | 1.1 | 3.6 |
|  | Marco | 1.0 | 0.6 | 1.8 | 2.2 |
|  | Marco | 0.4 | 0.2 | 1.1 | 1.0 |
|  | Marco | 0.7 | 0.5 | 1.1 | 3.9 |
|  | Marco | 0.8 | 0.5 | 1.2 | 3.8 |
|  | Marco | 1.1 | 0.7 | 1.6 | 3.9 |
|  | Marco | 1.0 | 0.7 | 1.5 | 4.6 |
|  | Marco | 1.1 | 0.6 | 1.8 | 2.7 |
|  | Marco | 0.6 | 0.3 | 1.2 | 1.6 |
|  | Marco | 1.0 | 0.7 | 1.5 | 4.7 |
|  | **Pooled OR** | 0.7 | 0.6 | 0.8 | 100.0 |
| ***Spring*** | Staceviciene | 1.0 | 0.5 | 1.7 | 7.0 |
|  | Baquero | 0.6 | 0.4 | 0.8 | 23.7 |
|  | Vardhan | 0.6 | 0.4 | 1.0 | 10.6 |
|  | Hoberman | 1.7 | 0.7 | 3.8 | 3.5 |
|  | Tam | 0.6 | 0.2 | 1.8 | 2.2 |
|  | Marco | 0.7 | 0.4 | 1.1 | 9.6 |
|  | Marco | 0.7 | 0.4 | 1.1 | 8.9 |
|  | Marco | 0.7 | 0.5 | 1.1 | 11.9 |
|  | Marco | 1.0 | 0.6 | 1.8 | 6.8 |
|  | Marco | 0.4 | 0.2 | 1.1 | 2.9 |
|  | Marco | 0.7 | 0.5 | 1.1 | 13.1 |
|  | **Pooled OR** | 0.7 | 0.6 | 0.8 | 100.0 |
| ***Summer*** | Staceviciene | 0.4 | 0.2 | 1.1 | 4.3 |
|  | Baquero | 0.9 | 0.6 | 1.4 | 14.2 |
|  | Vardhan | 0.4 | 0.2 | 0.7 | 7.6 |
|  | Hoberman | 0.4 | 0.0 | 3.5 | 0.9 |
|  | Tam | 0.5 | 0.2 | 1.5 | 3.1 |
|  | Marco | 0.8 | 0.5 | 1.2 | 12.5 |
|  | Marco | 1.1 | 0.7 | 1.6 | 12.8 |
|  | Marco | 1.0 | 0.7 | 1.5 | 14.1 |
|  | Marco | 1.1 | 0.6 | 1.8 | 9.9 |
|  | Marco | 0.6 | 0.3 | 1.2 | 6.4 |
|  | Marco | 1.0 | 0.7 | 1.5 | 14.4 |
|  | **Pooled OR** | 0.8 | 0.7 | 1.0 | 100.0 |
| ***Autumn*** | Staceviciene | 0.8 | 0.4 | 1.4 | 4.9 |
|  | Baquero | 0.6 | 0.4 | 0.8 | 15.9 |
|  | Vardhan | 0.5 | 0.3 | 0.9 | 6.3 |
|  | Hoberman | 0.4 | 0.1 | 1.5 | 0.9 |
|  | Tam | 0.7 | 0.3 | 2.0 | 1.7 |
|  | Marco | 0.6 | 0.4 | 0.8 | 12.6 |
|  | Marco | 0.6 | 0.4 | 0.9 | 11.7 |
|  | Marco | 0.6 | 0.5 | 0.9 | 15.6 |
|  | Marco | 0.7 | 0.4 | 1.1 | 7.7 |
|  | Marco | 0.7 | 0.4 | 1.2 | 5.8 |
|  | Marco | 0.7 | 0.5 | 0.9 | 17.1 |
|  | **Pooled OR** | 0.6 | 0.6 | 0.7 | 100.0 |

1. **Meta-analysis of studies done in the Northern hemisphere describing the seasonality of AMR rates in *S. pneumoniae* isolates to penicillins, winter as reference group.**

| **Season** | **Study** | **OR** | **95% CI** | | **% Weight** |
| --- | --- | --- | --- | --- | --- |
|  |  |  | **Lower** | **Upper** |  |
| ***All seasons*** | Baquero | 0.6 | 0.4 | 0.8 | 9.1 |
|  | Baquero | 0.6 | 0.4 | 0.8 | 8.8 |
|  | Baquero | 0.9 | 0.6 | 1.4 | 6.9 |
|  | Vardhan | 0.6 | 0.4 | 1.0 | 5.0 |
|  | Vardhan | 0.5 | 0.3 | 0.9 | 4.3 |
|  | Vardhan | 0.4 | 0.2 | 0.7 | 3.1 |
|  | Hoberman | 1.7 | 0.7 | 3.8 | 1.9 |
|  | Hoberman | 0.4 | 0.0 | 3.5 | 0.3 |
|  | Hoberman | 0.4 | 0.1 | 1.5 | 0.7 |
|  | Tam | 0.7 | 0.3 | 2.0 | 1.3 |
|  | Tam | 0.6 | 0.2 | 1.8 | 1.2 |
|  | Tam | 0.5 | 0.2 | 1.5 | 1.1 |
|  | Marco | 0.6 | 0.4 | 0.8 | 7.4 |
|  | Marco | 0.6 | 0.4 | 0.9 | 7.1 |
|  | Marco | 0.6 | 0.5 | 0.9 | 8.7 |
|  | Marco | 0.7 | 0.4 | 1.1 | 4.6 |
|  | Marco | 0.7 | 0.4 | 1.1 | 4.4 |
|  | Marco | 0.7 | 0.5 | 1.1 | 5.5 |
|  | Marco | 0.8 | 0.5 | 1.2 | 5.8 |
|  | Marco | 1.1 | 0.7 | 1.6 | 6.0 |
|  | Marco | 1.0 | 0.7 | 1.5 | 6.8 |
|  | **Pooled OR** | 0.7 | 0.6 | 0.8 | 100.0 |
| ***Spring*** | Baquero | 0.6 | 0.4 | 0.8 | 33.7 |
|  | Vardhan | 0.6 | 0.4 | 1.0 | 15.1 |
|  | Hoberman | 1.7 | 0.7 | 3.8 | 4.9 |
|  | Tam | 0.6 | 0.2 | 1.8 | 3.2 |
|  | Marco | 0.7 | 0.4 | 1.1 | 13.6 |
|  | Marco | 0.7 | 0.4 | 1.1 | 12.7 |
|  | Marco | 0.7 | 0.5 | 1.1 | 16.9 |
|  | **Pooled OR** | 0.7 | 0.6 | 0.8 | 100.0 |
| ***Summer*** | Baquero | 0.9 | 0.6 | 1.4 | 21.2 |
|  | Vardhan | 0.4 | 0.2 | 0.7 | 12.4 |
|  | Hoberman | 0.4 | 0.0 | 3.5 | 1.5 |
|  | Tam | 0.5 | 0.2 | 1.5 | 5.3 |
|  | Marco | 0.8 | 0.5 | 1.2 | 19.1 |
|  | Marco | 1.1 | 0.7 | 1.6 | 19.4 |
|  | Marco | 1.0 | 0.7 | 1.5 | 21.1 |
|  | **Pooled OR** | 0.8 | 0.6 | 1.0 | 100.0 |
| ***Autumn*** | Baquero | 0.6 | 0.4 | 0.8 | 24.6 |
|  | Vardhan | 0.5 | 0.3 | 0.9 | 9.7 |
|  | Hoberman | 0.4 | 0.1 | 1.5 | 1.4 |
|  | Tam | 0.7 | 0.3 | 2.0 | 2.6 |
|  | Marco | 0.6 | 0.4 | 0.8 | 19.5 |
|  | Marco | 0.6 | 0.4 | 0.9 | 18.1 |
|  | Marco | 0.6 | 0.5 | 0.9 | 24.2 |
|  | **Pooled OR** | 0.6 | 0.5 | 0.7 | 100.0 |

1. **Meta-analysis of studies done in European region describing the seasonality of AMR rates in *S. pneumoniae* isolates to all antibiotics, winter as reference group.**

| **Season** | **Study** | **OR** | **95% CI** | | **% Weight** |
| --- | --- | --- | --- | --- | --- |
|  |  |  | **Lower** | **Upper** |  |
| ***All seasons*** | Staceviciene | 0.8 | 0.4 | 1.4 | 2.3 |
|  | Staceviciene | 1.0 | 0.5 | 1.7 | 2.3 |
|  | Staceviciene | 0.4 | 0.2 | 1.1 | 1.0 |
|  | Baquero | 0.6 | 0.4 | 0.8 | 6.5 |
|  | Baquero | 0.6 | 0.4 | 0.8 | 6.3 |
|  | Baquero | 0.9 | 0.6 | 1.4 | 4.8 |
|  | Vardhan | 0.6 | 0.4 | 1.0 | 3.4 |
|  | Vardhan | 0.5 | 0.3 | 0.9 | 2.9 |
|  | Vardhan | 0.4 | 0.2 | 0.7 | 2.1 |
|  | Marco | 0.6 | 0.4 | 0.8 | 5.2 |
|  | Marco | 0.6 | 0.4 | 0.9 | 4.9 |
|  | Marco | 0.6 | 0.5 | 0.9 | 6.2 |
|  | Marco | 0.7 | 0.4 | 1.1 | 3.5 |
|  | Marco | 0.7 | 0.4 | 1.2 | 2.7 |
|  | Marco | 0.7 | 0.5 | 0.9 | 6.6 |
|  | Marco | 0.7 | 0.4 | 1.1 | 3.1 |
|  | Marco | 0.7 | 0.4 | 1.1 | 2.9 |
|  | Marco | 0.7 | 0.5 | 1.1 | 3.8 |
|  | Marco | 1.0 | 0.6 | 1.8 | 2.3 |
|  | Marco | 0.4 | 0.2 | 1.1 | 1.0 |
|  | Marco | 0.7 | 0.5 | 1.1 | 4.1 |
|  | Marco | 0.8 | 0.5 | 1.2 | 4.0 |
|  | Marco | 1.1 | 0.7 | 1.6 | 4.1 |
|  | Marco | 1.0 | 0.7 | 1.5 | 4.8 |
|  | Marco | 1.1 | 0.6 | 1.8 | 2.9 |
|  | Marco | 0.6 | 0.3 | 1.2 | 1.6 |
|  | Marco | 1.0 | 0.7 | 1.5 | 4.9 |
|  | **Pooled OR** | 0.7 | 0.6 | 0.8 | 100.0 |
| ***Spring*** | Staceviciene | 1.0 | 0.5 | 1.7 | 7.4 |
|  | Baquero | 0.6 | 0.4 | 0.8 | 25.1 |
|  | Vardhan | 0.6 | 0.4 | 1.0 | 11.2 |
|  | Marco | 0.7 | 0.4 | 1.1 | 10.2 |
|  | Marco | 0.7 | 0.4 | 1.1 | 9.5 |
|  | Marco | 0.7 | 0.5 | 1.1 | 12.6 |
|  | Marco | 1.0 | 0.6 | 1.8 | 7.2 |
|  | Marco | 0.4 | 0.2 | 1.1 | 3.1 |
|  | Marco | 0.7 | 0.5 | 1.1 | 13.9 |
|  | **Pooled OR** | 0.7 | 0.6 | 0.8 | 100.0 |
| ***Summer*** | Staceviciene | 0.4 | 0.2 | 1.1 | 4.6 |
|  | Baquero | 0.9 | 0.6 | 1.4 | 14.5 |
|  | Vardhan | 0.4 | 0.2 | 0.7 | 8.2 |
|  | Marco | 0.8 | 0.5 | 1.2 | 13.0 |
|  | Marco | 1.1 | 0.7 | 1.6 | 13.2 |
|  | Marco | 1.0 | 0.7 | 1.5 | 14.5 |
|  | Marco | 1.1 | 0.6 | 1.8 | 10.4 |
|  | Marco | 0.6 | 0.3 | 1.2 | 6.9 |
|  | Marco | 1.0 | 0.7 | 1.5 | 14.7 |
|  | **Pooled OR** | 0.8 | 0.7 | 1.0 | 100.0 |
| **Autumn** | Staceviciene | 0.8 | 0.4 | 1.4 | 5.0 |
|  | Baquero | 0.6 | 0.4 | 0.8 | 16.3 |
|  | Vardhan | 0.5 | 0.3 | 0.9 | 6.4 |
|  | Marco | 0.6 | 0.4 | 0.8 | 12.9 |
|  | Marco | 0.6 | 0.4 | 0.9 | 12.0 |
|  | Marco | 0.6 | 0.5 | 0.9 | 16.0 |
|  | Marco | 0.7 | 0.4 | 1.1 | 7.9 |
|  | Marco | 0.7 | 0.4 | 1.2 | 6.0 |
|  | Marco | 0.7 | 0.5 | 0.9 | 17.6 |
|  | **Pooled OR** | 0.6 | 0.6 | 0.7 | 100.0 |

1. **Meta-analysis of studies done in European region describing the seasonality of AMR rates in *S. pneumoniae* isolates to all penicillins, winter as reference group.**

| **Season** | **Study** | **OR** | **95% CI** | | **% Weight** |
| --- | --- | --- | --- | --- | --- |
|  |  |  | **Lower** | **Upper** |  |
| ***All seasons*** | Baquero | 0.6 | 0.4 | 0.8 | 9.6 |
|  | Baquero | 0.6 | 0.4 | 0.8 | 9.3 |
|  | Baquero | 0.9 | 0.6 | 1.4 | 7.4 |
|  | Vardhan | 0.6 | 0.4 | 1.0 | 5.4 |
|  | Vardhan | 0.5 | 0.3 | 0.9 | 4.7 |
|  | Vardhan | 0.4 | 0.2 | 0.7 | 3.4 |
|  | Marco | 0.6 | 0.4 | 0.8 | 7.9 |
|  | Marco | 0.6 | 0.4 | 0.9 | 7.5 |
|  | Marco | 0.6 | 0.5 | 0.9 | 9.2 |
|  | Marco | 0.7 | 0.4 | 1.1 | 5.0 |
|  | Marco | 0.7 | 0.4 | 1.1 | 4.7 |
|  | Marco | 0.7 | 0.5 | 1.1 | 5.9 |
|  | Marco | 0.8 | 0.5 | 1.2 | 6.2 |
|  | Marco | 1.1 | 0.7 | 1.6 | 6.4 |
|  | Marco | 1.0 | 0.7 | 1.5 | 7.3 |
|  | **Pooled OR** | 0.7 | 0.6 | 0.8 | 100.0 |
| ***Spring*** | Baquero | 0.6 | 0.4 | 0.8 | 36.6 |
|  | Vardhan | 0.6 | 0.4 | 1.0 | 16.4 |
|  | Marco | 0.7 | 0.4 | 1.1 | 14.8 |
|  | Marco | 0.7 | 0.4 | 1.1 | 13.8 |
|  | Marco | 0.7 | 0.5 | 1.1 | 18.4 |
|  | **Pooled OR** | 0.6 | 0.5 | 0.8 | 100.0 |
| ***Summer*** | Baquero | 0.9 | 0.6 | 1.4 | 22.5 |
|  | Vardhan | 0.4 | 0.2 | 0.7 | 13.9 |
|  | Marco | 0.8 | 0.5 | 1.2 | 20.5 |
|  | Marco | 1.1 | 0.7 | 1.6 | 20.8 |
|  | Marco | 1.0 | 0.7 | 1.5 | 22.4 |
|  | **Pooled OR** | 0.8 | 0.6 | 1.1 | 100.0 |
| **Autumn** | Baquero | 0.6 | 0.4 | 0.8 | 25.7 |
|  | Vardhan | 0.5 | 0.3 | 0.9 | 10.1 |
|  | Marco | 0.6 | 0.4 | 0.8 | 20.3 |
|  | Marco | 0.6 | 0.4 | 0.9 | 18.8 |
|  | Marco | 0.6 | 0.5 | 0.9 | 25.2 |
|  | **Pooled OR** | 0.6 | 0.5 | 0.7 | 100.0 |
