# Supplementary material for: Seasonality of antimicrobial resistance rates in respiratory bacteria: A systematic review and meta-analysis
Source: PLoS One. 2019 Aug 15;14(8):e0221133. doi: 10.1371/journal.pone.0221133 (PMC6695168; doi:10.1371/journal.pone.0221133)
Supplement: S5 Table — Egger´s test. (DOCX) [file pone.0221133.s007.docx]

# S5 Table. Assessment of publication bias. Egger´s test

| **N**  **Studies** | **Antibiotic class** | **Hemisphere** | **Region** | **Season comparison** | | **Egger´s test (Bias effect)** | | | |
| --- | --- | --- | --- | --- | --- | --- | --- | --- | --- |
|  |  |  |  | **Season** | **Ref.** | **Coef.** | **Se** | **t** | **P_value** |
| ***Streptococcus pneumoniae*** | | | | | | | | | |
| 7 | PEN, MC, CEP, TM/SUL, MDR | N, N/W, N/E, S | EU, AM,EA | All seasons | Winter | -0,84 | 0,30 | -2,74 | 0,001* |
| 6 | PEN, MC, CEP, TM/SUL, MDR | N, N/W, N/E, S | EU, AM,EA | Spring | Winter | 1,38 | 0,83 | 1,65 | 0,131 |
| 7 | PEN, MC, CEP, TM/SUL, MDR | N, N/W, N/E, S | EU, AM,EA | Summer | Winter | -1,38 | 0,60 | -2,30 | 0,042* |
| 6 | PEN, MC, CEP, TM/SUL, MDR | N, N/W, N/E, S | EU, AM,EA | Autumn | Winter | -0,33 | 0,41 | -0,81 | 0,440 |
| 6 | PEN | N, N/W, N/E, S | EU, AM,EA | All seasons | Winter | -0,67 | 0,38 | -1,74 | 0,089 |
| 5 | PEN | N, N/W, N/E, S | EU, AM,EA | Spring | Winter | 1,96 | 0,78 | 2,52 | 0,045* |
| 6 | PEN | N, N/W, N/E, S | EU, AM,EA | Summer | Winter | -1,10 | 0,72 | -1,54 | 0,168 |
| 5 | PEN | N, N/W, N/E, S | EU, AM,EA | Autumn | Winter | -0,57 | 0,34 | -1,65 | 0,151 |
| 6 | PEN, MC, CEP, TM/SUL, MDR | N | EU, AM,EA | All seasons | Winter | -0,53 | 0,39 | -1,34 | 0,185 |
| 6 | PEN, MC, CEP, TM/SUL, MDR | N | EU, AM,EA | Spring | Winter | 1,38 | 0,83 | 1,65 | 0,131 |
| 6 | PEN, MC, CEP, TM/SUL, MDR | N | EU, AM,EA | Summer | Winter | -1,64 | 0,68 | -2,37 | 0,039* |
| 6 | PEN, MC, CEP, TM/SUL, MDR | N | EU, AM,EA | Autumn | Winter | -0,33 | 0,41 | -0,81 | 0,440 |
| 5 | PEN | N | EU, AM,EA | All seasons | Winter | -0,42 | 0,47 | -0,90 | 0,374 |
| 5 | PEN | N | EU, AM,EA | Spring | Winter | 1,96 | 0,77 | 2,52 | 0,045* |
| 5 | PEN | N | EU, AM,EA | Summer | Winter | -1,34 | 0,87 | -1,53 | 0,178 |
| 5 | PEN | N | EU, AM,EA | Autumn | Winter | -0,57 | 0,34 | -1,65 | 0,151 |
| 3 | PEN,CEP, MDR | N | EU | All seasons | Winter | -0,38 | 0,38 | -1,01 | 0,316 |
| 3 | PEN,CEP, MDR | N | EU | Spring | Winter | 1,36 | 0,87 | 1,55 | 0,155 |
| 3 | PEN,CEP, MDR | N | EU | Summer | Winter | -1,35 | 0,45 | -2,97 | 0,016* |
| 3 | PEN,CEP, MDR | N | EU | Autumn | Winter | -0,24 | 0,4 | -0,6 | 0,563 |
| 2 | PEN | N | EU | All seasons | Winter | -0,23 | 0,45 | -0,53 | 0,603 |
| 2 | PEN | N | EU | Spring | Winter | 1,96 | 0,84 | 2,32 | 0,068 |
| 2 | PEN | N | EU | Summer | Winter | -0,94 | 0,42 | -2,23 | 0,076 |
| 2 | PEN | N | EU | Autumn | Winter | -0,49 | 0,34 | -1,44 | 0,211 |
| 8 | PEN, MC, CEP, TM/SUL, MDR | N | EU, AM,EA | Autumn | Spring | -0,46 | 0,33 | -1,37 | 0,182 |
| 7 | PEN, MC, CEP, TM/SUL, MDR | N | EU | Autumn | Spring | -0,13 | 0,27 | -0,49 | 0,630 |
| 8 | PEN | N | EU, AM,EA | Autumn | Spring | -0,64 | 0,39 | -1,64 | 0,116 |
| 7 | PEN | N | EU | Autumn | Spring | -0,27 | 0,29 | -0,95 | 0,356 |

*= significant publication bias P>0.05. PEN = penicillins, MC= macrolides, CEP= cephalosporins, TM/SUL = combination of trimethoprim/ sulfaminde, MDR = Multidrug-resistant, . EU= Europe, AM = America, WP= Wester Pacific, EA= East Asian
