# Supplementary material for: Seasonality of antimicrobial resistance rates in respiratory bacteria: A systematic review and meta-analysis
Source: PLoS One. 2019 Aug 15;14(8):e0221133. doi: 10.1371/journal.pone.0221133 (PMC6695168; doi:10.1371/journal.pone.0221133)
Supplement: S1 Fig — (DOCX) [file pone.0221133.s008.docx]

# S1 Fig. Sensitivity analysis forest-plot


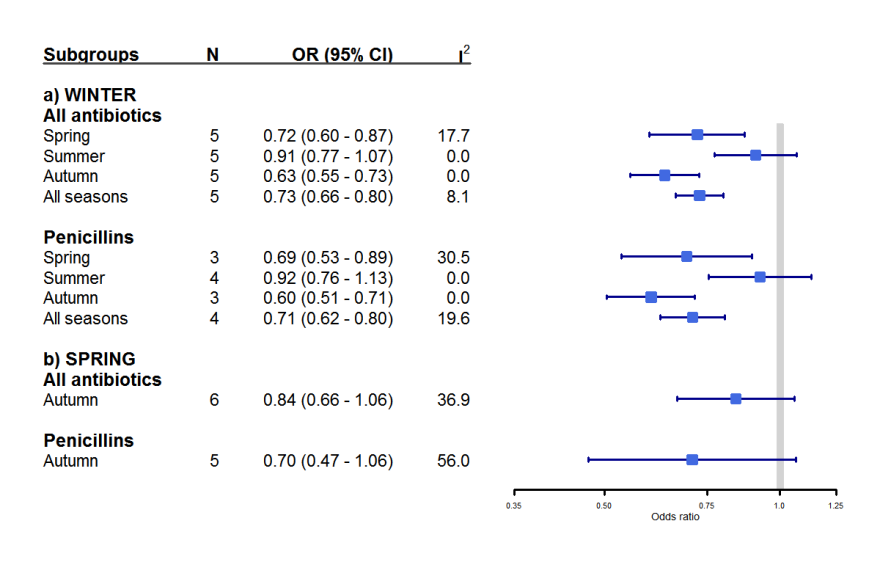


**Sensitivity analysis forest plot** **of seasonality of antimicrobial resistance rates in *Streptococcus pneumoniae* isolates**. Analysis was done by excluding two studies (Vardhan & Allen, 2003; Tam et al. 2015) in which the denominator for seasonal resistance rates was imputed. Studies were stratified into two subgroups of antibiotics and estimates of effect are presented as pooled odds ratios (squares) with 95% confidence intervals (lateral lines of squares). For comparison, winter and spring were the reference groups, thus equal to one. Solid vertical line limits no difference between the two groups. I^2^ refers to percentage of heterogeneity among studies. The “All antibiotics” subgroup includes penicillins, cephalosporins, macrolides, trimethoprim/sulphamides and multi-drug resistance.
