# Supplementary material for: Seasonality of antimicrobial resistance rates in respiratory bacteria: A systematic review and meta-analysis
Source: PLoS One. 2019 Aug 15;14(8):e0221133. doi: 10.1371/journal.pone.0221133 (PMC6695168; doi:10.1371/journal.pone.0221133)
Supplement: S2 Fig — (DOCX) [file pone.0221133.s009.docx]

# S2 Fig. Funnel plots

1. **Funnel plots of *S. pneumoniae* meta-analysis independently of study region and antibiotic class.**


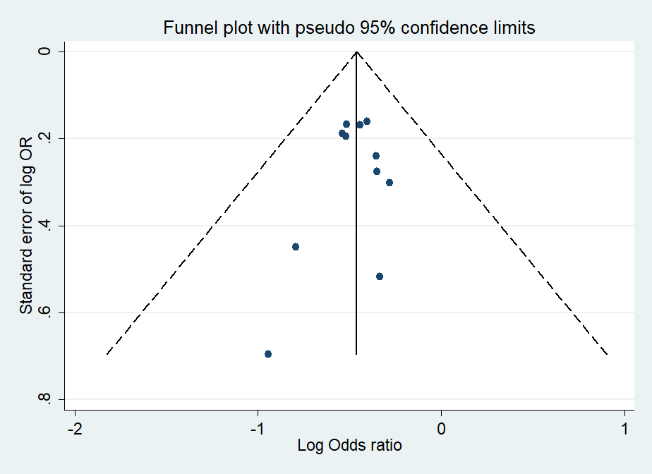


1. Funnel plot of the meta-analysis of studies with penicillin-resistant *S. pneumoniae* isolates. Autumn vs winter comparison.


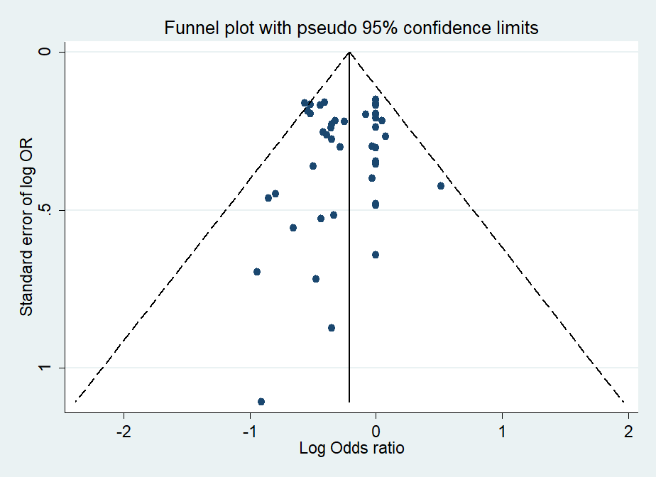


1. Funnel plot of the meta-analysis of studies with penicillin-resistant *S. pneumoniae* isolates. All seasons vs winter comparison.


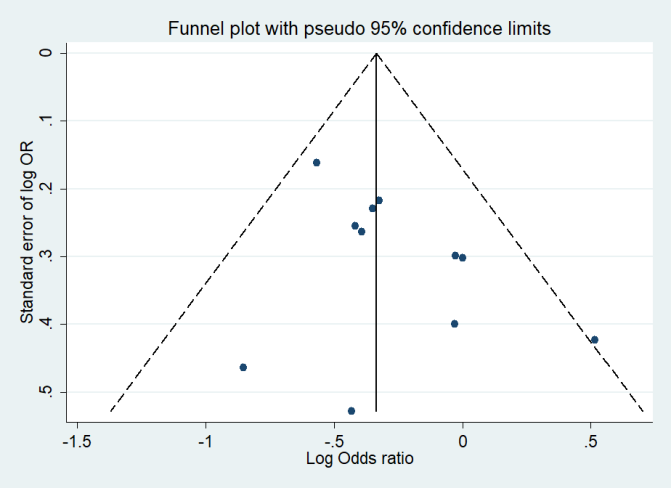

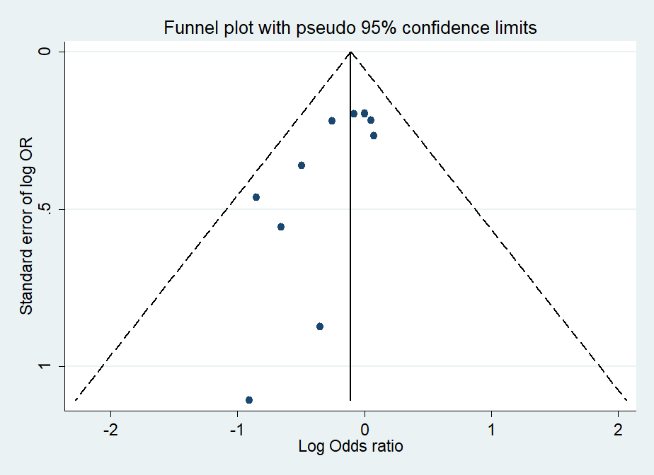


1. Funnel plot of the meta-analysis of studies with penicillin-resistant *S. pneumoniae* isolates. Summer vs winter comparison.
2. Funnel plot of the meta-analysis of studies with penicillin-resistant *S. pneumoniae* isolates. Spring vs winter comparison.

**B) Funnel plots of *S.pneumoniae* meta-analysis independently of study region and only comparing isolates resistant to penicillins**


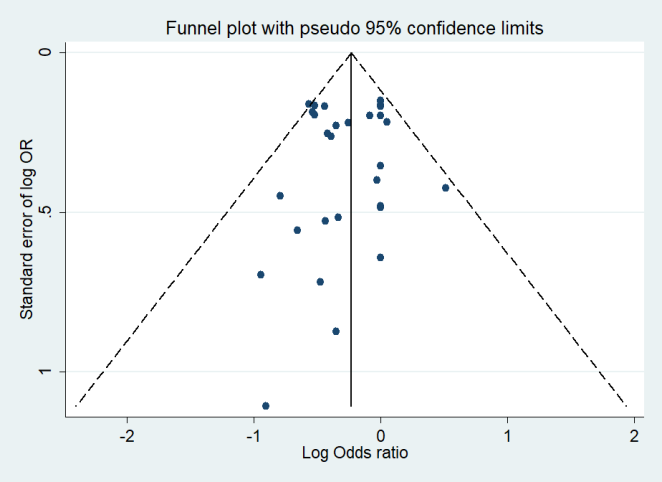

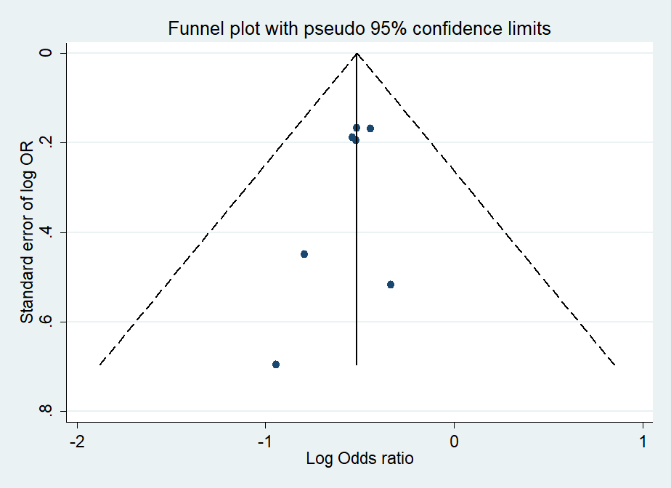


1. Funnel plot of the meta-analysis of studies with penicillin-resistant *S. pneumoniae* isolates. All seasons vs winter comparison.
2. Funnel plot of the meta-analysis of studies with penicillin-resistant *S. pneumoniae* isolates. Autumn vs winter comparison.


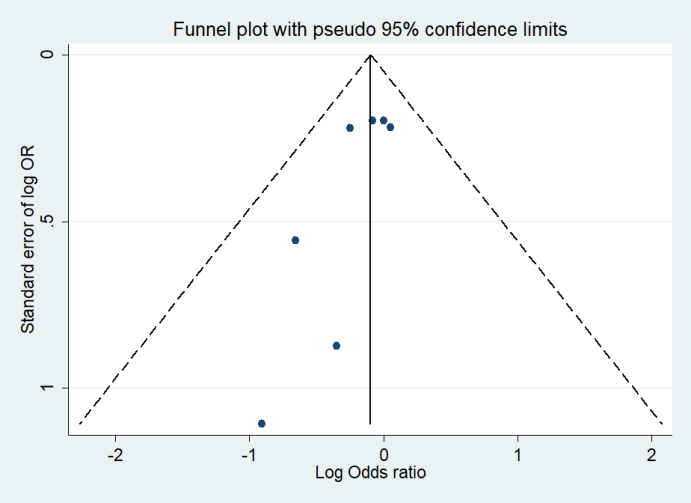


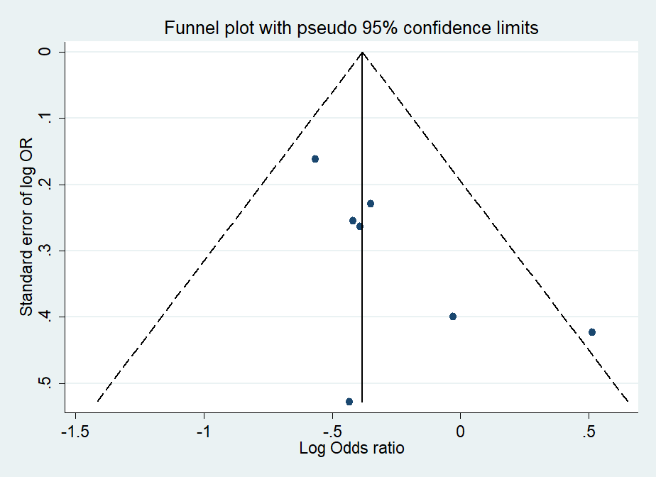


1. Funnel plot of the meta-analysis of studies with penicillin-resistant *S. pneumoniae* isolates. Summer vs winter comparison.
2. Funnel plot of the meta-analysis of studies with penicillin-resistant *S. pneumoniae* isolates. Spring vs winter comparison.
